# A Corticothalamic Circuit Model for Sound Identification in Complex Scenes

**DOI:** 10.1371/journal.pone.0024270

**Published:** 2011-09-13

**Authors:** Gonzalo H. Otazu, Christian Leibold

**Affiliations:** 1 Division of Neurobiology, Department Biology II, Ludwig-Maximilians-Universität, Munich, Germany; 2 Bernstein Center for Computational Neuroscience, Munich, Germany; Indiana University, United States of America

## Abstract

The identification of the sound sources present in the environment is essential for the survival of many animals. However, these sounds are not presented in isolation, as natural scenes consist of a superposition of sounds originating from multiple sources. The identification of a source under these circumstances is a complex computational problem that is readily solved by most animals. We present a model of the thalamocortical circuit that performs level-invariant recognition of auditory objects in complex auditory scenes. The circuit identifies the objects present from a large dictionary of possible elements and operates reliably for real sound signals with multiple concurrently active sources. The key model assumption is that the activities of some cortical neurons encode the difference between the observed signal and an internal estimate. Reanalysis of awake auditory cortex recordings revealed neurons with patterns of activity corresponding to such an error signal.

## Introduction

**Figure 1:**
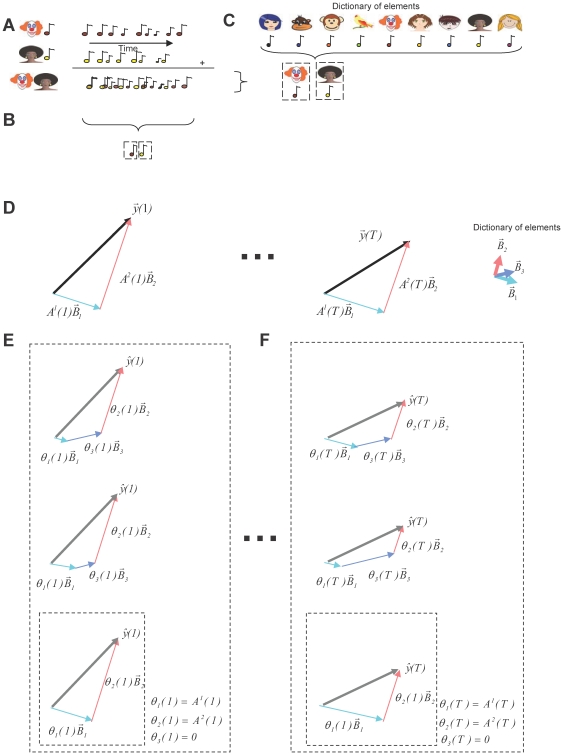
Identification of sources present in complex auditory scenes using large dictionaries. (**A**) Auditory scenes are composed of sounds generated by different sources. (**B**) Blind source separation methods estimate the sources present in a scene based only on the observed scene. (**C**) Other algorithms assume that the sources present in a scene are part of a very large dictionary of possible sources (represented by the collection of pictures and the associated sounds). (**D**) These algorithms also assume that the sources present are combined linearly, (vectors 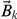 multiplied by the time varying amplitude 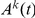), to generate the time varying scene (time-varying vector 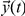). (**E**) Algorithms as in **D** create an estimate of the observed signal by combining elements of the dictionary, each one weighted by an time varying estimated parameter 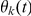. For large dictionaries, there are multiple estimated parameters that create an estimate of the observation that matches equally well to the observed signal (represented by the different combinations of vectors inside the large square that generate the same well-matched estimate 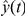). A single solution is chosen by minimizing the number of active dictionary elements (vector combination inside the smaller square). (**F**) At each time step, a new set of parameters 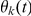 is estimated that reflect the contribution of the identified dictionary element to the current auditory scene 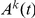. The other estimated parameters 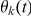 are zero.

Auditory scenes are generally composed of sounds produced by multiple sources. The observed complex auditory signal is a superposition of these sources, making the identification of the individual sound elements a non-trivial problem (**Fig. 1A**). While humans generally perform better than machines do in recognizing auditory objects in complex scenes, it is not yet known how our nervous system performs this task in real time.

In one family of computer algorithms, the blind source separation algorithms (**Fig. 1B**), source elements are identified using only the information extracted from the observed signal. These approaches make no parametric assumptions about the superimposed signals in the auditory scene. Without such prior information, the amount of data necessary to identify the sources present in a scene is large, making them not compatible with the real time requirement for biological systems.

An alternative family of computer algorithms assumes that the elements that are present in a scene belong to a large, but finite, dictionary of known sounds (**Fig. 1C**) [1], [2], [3], [4], [5], [6]. By making this assumption, the number of observations required to identify a source is substantially reduced, making them more suitable for biological systems. These algorithms assume that the observed auditory scene originated by a time-varying linear combination of just a few elements that belonged to the dictionary (**Fig. 1D**). Then, the dictionary elements are selected such that an appropriate linear combination would reconstruct the observed signal with the highest fidelity. As the elements present in an auditory scene have to be part of the dictionary, these algorithms require a very large number of dictionary elements. However, if the dictionary is large enough, there are multiple combinations of elements that would reconstruct the observed signal with the same high fidelity (**Fig. 1E–F**). To enforce uniqueness of the solution, those algorithms require additional minimization of a secondary cost function. Since a typical auditory scene is composed of only few elements, this secondary objective is taken as the number of active dictionary elements. Due to this additional cost function the number of identified dictionary elements is small, and therefore these algorithms provide possible models for sparse codes in sensory brain regions [7].

A particular auditory scene activates only a few from the large number of neurons available in the auditory cortex [8], [9], [10], [11], [12], which matches the behavior of sparse coding algorithms. However, we do not know which of these algorithms the auditory system really implements, and what are the mechanisms the brain uses to select the dictionary elements that are present in a scene.

In this paper, we propose a new dictionary-based algorithm, the **C**orrected **P**rojections **A**lgorithm (CPA). It only uses the minimization of the difference between the sensory representation of the incoming sound and an internal estimate to identify the sources present in the auditory scene. CPA does not explicitly minimize the number of active dictionary elements; the sparse representation is a direct consequence of the model design. The estimated parameters indicate the presence or absence of a particular dictionary element and its behavior matches certain aspects of the psychophysics of auditory stream perception. Here, we propose the hypothesis that the architecture of the corticothalamic circuit matches an efficient circuit implementation of CPA, and we show cortical recordings that are consistent with the proposed role of auditory cortex in the implementation of CPA.

## Results

### CPA works on superimposed sound sources

CPA uses similar assumptions as previous source identification algorithms (**Fig. 1D**), mainly that the stimulus originated from linear combination of the sources [1], [2], [3], [4], [5], [6]. For illustrative purposes, we will assume an auditory scene where each sound source *k* has a characteristic frequency spectrum 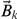, which is stationary over time [13]. For non-stationary spectra the feature vectors 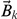 may also be generalized to the spectrotemporal domain, or even to include higher order cues of complex signals like speech. The shape of the spectrum originating from each source is assumed to be stationary, but the amplitude 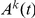 fluctuates in time (**Text S1: Definition of auditory scene**), that is at any instant *t*, the superposition of several of these sources creates the observed signal 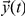.
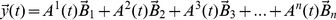
(1)


**Figure 2:**
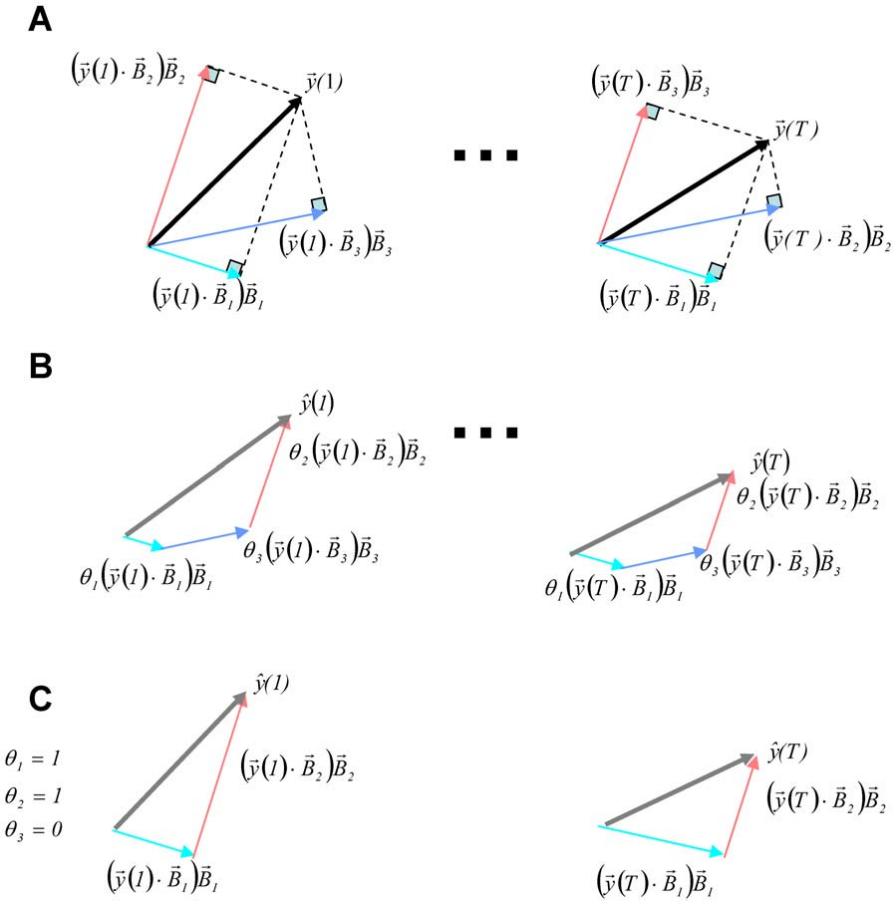
The Corrected Projections Algorithm (CPA). (**A**) At each time step, CPA calculates the projections 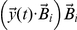 of the observed signal 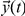 onto all the dictionary elements 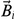. (**B**) CPA uses the linear combination of all projections 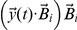, each combined with a non-time varying presence parameter 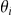, to generate an estimate 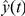 of the signal 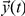. (**C**) The minimization of the minimal square error between all T observations 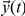 and the T estimates 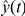 yields a single solution for the presence parameters 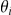. The elements that are present in a scene are indicated by a value of one and absent elements are marked with a value of zero.

We assume that each source generates its sounds independently of other sources. Therefore, the amplitude modulations 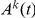 of the different objects are uncorrelated. We assume that the system has previously learned a large dictionary of *n* possible sound elements 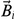 (*n>>f*, where *f* is the number of features of the signal 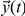, in this case, *f* will be the number of frequency bands of the spectrum) that includes the sources 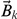 that generated the signal 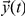. CPA receives as input *T* samples of the observed signal 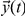 and, based on temporal fluctuations of the contributing dictionary elements [14], outputs a unique set of parameters 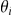, *i = 1..n* (Fig. **2**). The parameters 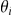 estimated by CPA will take the value of one, if the corresponding dictionary element is part of the auditory scene, or zero, if it is not.

### CPA finds a unique set of presence parameters by minimizing estimation error

Similar to previous algorithms [1], [2], [3], [4], [5], [6], CPA creates an estimate 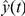 about the current auditory scene and determines the parameters 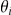 by finding the set of parameters that minimize the square error between the estimate 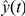 and the observed signal 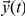. Previous algorithms' estimates 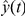 had the same structure as the model of the auditory scene, mainly (cf. **equation 1**)
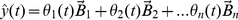
(2)


There are two main differences of CPA with such previous algorithms. The first one is the way the estimate 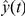 is generated. The CPA estimate is
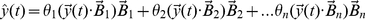
(3)Each dictionary element is additionally weighted with its similarity 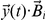; forming the time-varying projection of the vector 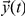 onto the vector 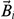 (**Fig. 2A** and **Text S2: Corrected projections algorithm**). The second difference is in the way that multiple observations are processed. In the standard algorithms, the estimated parameters 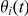 are generated at the sampling rate of the input. In contrast, CPA estimates a single set of parameters 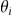 for all *T* observed samples (**Fig. 2B**). CPA estimated parameters do not indicate the instantaneous contribution of a dictionary element, but its presence or absence in an auditory scene (**Fig. 2C**). Therefore, we called the CPA parameters 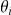 presence parameters.

The inclusion of the similarities 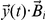 in the estimate 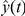 is the key element that causes this minimization to yield a unique set of presence parameters 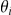, without requiring any additional constraints (**Text S2: Corrected projections algorithm**). This uniqueness property of CPA contrasts to other algorithms that require additional constraints to find unique solutions [1], [2], [3], [4], [5], [6].

### CPA presence parameters are binary variables that indicate the presence of known sound sources in a scene

**Figure 3:**
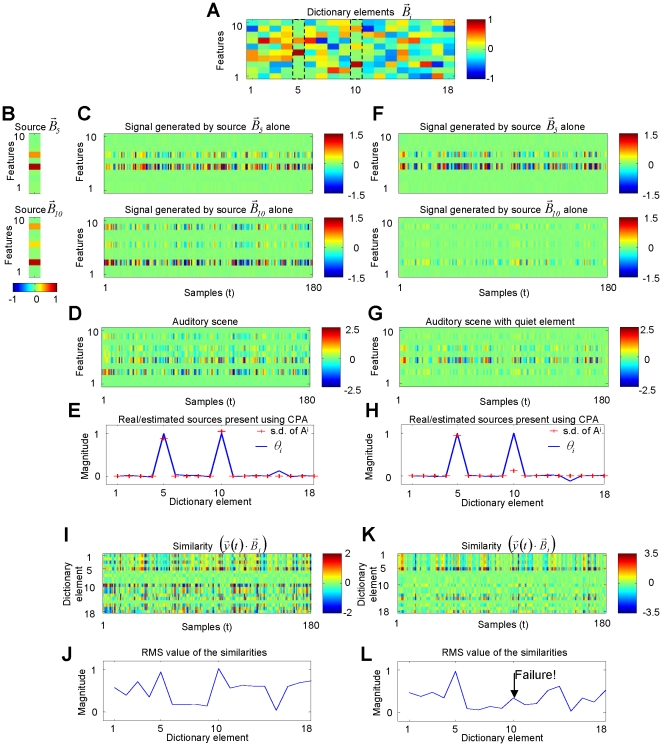
CPA identifies overlapping sources. (**A**) Two elements 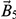 and 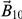 were selected from a dictionary of 18 elements. (**B–C–D**) Each one of these elements were amplitude modulated and the amplitude modulated signals were added to create the auditory scene 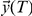. The signals were equally loud. (**E**) CPA is able to identify the two dictionary elements that generated the signal by a large magnitude in the respective presence parameters. (**F–G**) The elements from the dictionary were again used to generate an auditory scene, but with the amplitude of one of the elements reduced 10-fold. (**H**) CPA still correctly detected the presence of the quieter element. (**I–J–K–L**) **CPA outperforms template matching**. (**I–J**) For the case where the elements are equally loud (case **C–D**), the two elements present could have been identified as the most similar ones to the observed signal and they could have been detected as the peak values in the RMS of the similarities. (**K–L**) This method failed in the case of a quiet element (**F–G**), as there were other elements with larger root-mean-square similarity than the quiet element.

If signals that match the sources in the scene are part of the dictionary (**Fig. 3A**), and if the sources present 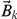 are orthogonal, minimizing the average error will identify sources present in a scene by finding the correct set of the presence parameters 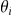 (**Fig. 3B–E**). A correct identification consists of 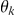 *= 1* for each one of the few sources participating in the scene and 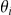 *= 0* for the large number of dictionary elements that are not part of the scene (**Text S3: Proof that CPA detects the elements present in a mixture**). This typical binary behavior of the presence parameters 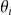 makes apparent that CPA works as a recognition algorithm, meaning it finds specific dictionary elements representing identified sources. Although the orthogonality requirement seems restrictive, it applies only to the dictionary elements 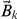 that are present in a particular scene and not to the whole dictionary, which would have limited the number of dictionary elements to the number of features of the dictionary elements. Moreover, CPA is robust to small deviations from orthogonality in the present sources 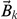, which is the case for most pairs of vectors if the number of features *f* of the input signal is large enough, potentially allowing the use of very large dictionaries (see **Text S4: Effects of auditory scene complexity and dictionary size on CPA performance** and **Fig. S1** for a case in which there is more overlap). If the sources in a scene can be represented by orthogonal elements, a common approach is to estimate them using Principal Component Analysis (PCA). However, PCA might require larger amounts of input data than CPA because PCA does not incorporate prior information from a dictionary (**Fig. S2**).

The presence parameters of CPA indicate the presence or absence of learned sound objects that are already part of the dictionary. An element that has not been encountered before will not be recognized, as there won't be a single presence parameter with a value of 1 indicating its participation in the scene. Instead, it will appear as small values over multiple presence parameters (**Fig. S3**).

### CPA estimated parameters are invariant to sound intensity

CPA still identifies the elements present (**Fig. 3F–H**), even if the contribution of one of the sources is quieter than the other sources. The presence parameters 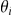 hence indicate the presence or absence of a source 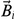, independent of the magnitude 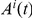 of the source's contribution to the auditory scene, for all observed *T* samples. This is different from previous algorithms [1], [2], [3], [4], [6], which would have yielded a time-varying parameters 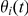 that indicates the instantaneous contribution of the corresponding dictionary element 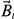 to the auditory scene at that moment in time.

### CPA solves problems that cannot be solved by template matching

The observed signal is generally not a good match to the respective dictionary elements that generated it because the sound of the scene is a superposition of multiple objects. A standard method for pattern recognition, template matching, identifies the sources present in a scene by calculating how similar the instantaneous spectrum 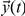 is to each of the *n* possible sound sources and identifying the sources present as the most similar ones. When the contributions of the present sources to the signal 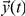 are equally large, we can recognize that the elements 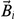 with the largest similarity 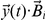 are the elements that participated in the mixture (**Fig. 3I–J**). However, the similarities give a more ambiguous picture of the elements present than the presence parameters of CPA (compare **Fig. 3E** with **Fig. 3J**). In the case where one of the elements is quieter than the other (**Fig. 3F–H**), the similarities fail to identify the more quiet source (**Fig. 3K–L**), as the observed scene is more similar to other dictionary elements that were not present than to the more quiet element that participated in the scene.

### Iterative implementation of CPA is computationally efficient

The original formulation of CPA is not a realistic model for the brain's sensory system because it requires storing all the *T* observations of an auditory scene. The original CPA also cannot handle dictionaries with large number *n* of elements because it requires the inversion of a square matrix of *n* dimensions, which is numerically ill- conditioned for large *n* (**Text S2: Corrected projections algorithm**). However, the fact that in CPA the minimization of the difference between the observed signal 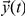 and the estimate of that signal 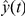 yields a single solution for 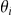, permits to use an efficient and numerically robust implementation, which is similar to a Kalman filter [15]. A similar implementation cannot be straight-forwardly generalized to previous algorithms [1], [2], [3], [4], [5], [6] because the minimization of the estimation error does not yield a unique solution for the parameters 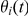.

**Figure 4:**
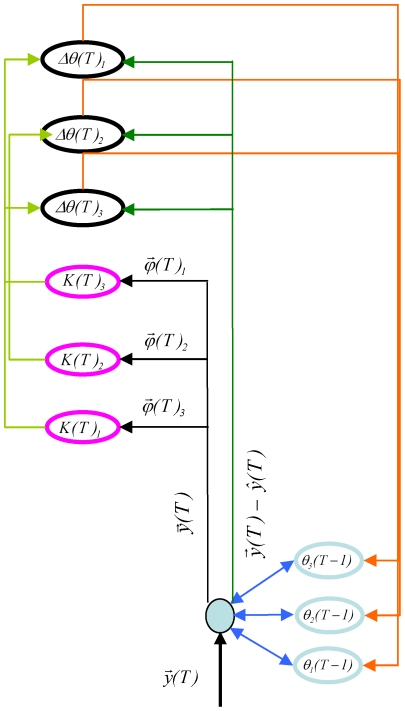
The architecture of the thalamocortical system matches an efficient iterative implementation of CPA. ICPA uses the previous estimate of the presence parameters 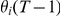 (light blue circles; corresponding to each one of the dictionary elements 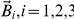) to generate an estimate 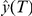 of the current stimulus 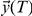. The error in the estimate 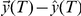 is converted into an error in the presence parameters 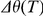 (black circles). This transformation requires a large population 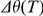 (displayed as the black circles) that tracks the error in the prediction for each dictionary element 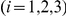 and another population ***K*** *(T)* (magenta circles) that represents the uncertainty of each of these elements, matching the expansion in number of cells seen in the cortex, compared to the number of inputs from the periphery. The population ***K*** *(T)* receives as input the projections 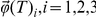 into the dictionary elements, which can be calculated from the current stimulus 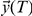. The error in the parameters 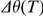 is sent via the massive thalamocortical feedback connection (orange) to be integrated into an updated parameter.

This efficient implementation of CPA, which we call **i**terative **CPA** or **iCPA** (**Fig. 4**), exploits the fact that sound samples 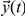 appear sequentially in time to reduce the memory requirements and computational complexity. Instead of storing all the observations of the incoming signal 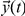 up to time *T-1*, it stores an internal estimate of the *n* presence parameters 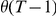 based on the past *T-1* samples. The previous parameters 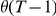 combined with the current projections 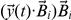 create a new estimate 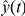, analogous to **Fig. 2B** and **equation 2**. The presence parameters 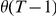 are updated proportionally to the *f*-dimensional difference between the incoming signal 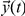 and its estimate 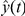,
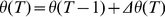
(4)where
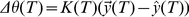
(5)is the *n*-dimensional error signal of the presence parameters. If the parameters 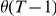 already generate an estimate 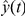 that is similar to the signal 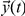, the parameters will not be updated. The error in the presence parameter 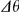 also depends on the *n by f* sensitivity matrix ***K*** *(T)*. ***K*** *(T)* represents the *uncertainty* about the stored presence parameters 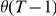. In case there is a large uncertainty about the presence parameters, ***K*** *(T)*, which depends on the dictionary elements 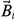 and the observed signal 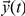, will have a large value. In this case, the presence parameters will be updated by a large amount, even for a small estimation error 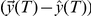.

**Figure 5:**
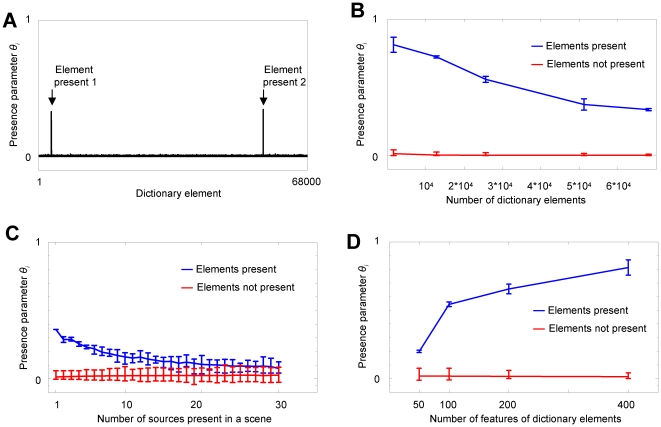
ICPA is a robust estimator. (**A**) iCPA identifies two random non-orthogonal sources of *f* = 400 features using a large dictionary of *n* = 68000 possible sources. (**B–D**) **Dependence of iCPA on number of elements present, in a scene, size of the dictionary, and number of features.** The values of the estimated presence parameters for the elements that generated the signal are shown in blue and for the elements that did not generate the signal are shown in red. The error bars indicate the full range of values. (**B**) iCPA can handle large number of dictionary elements. The figure was generated using 2 sources and a signal dimension of *f* = 400. (**C**) ICPA fails if the number of simultaneously present sources increase. The figure was generated using *f* = 100 and *n* = 3200 dictionary elements. (**D**) The performance of iCPA improves as the number *f* of features increases. The figure was generated using 2 sources present and a dictionary of *n* = 1600 elements.

ICPA is mathematically equivalent to the non-iterative CPA. However, iCPA is numerically better conditioned, because it requires the inversion of a much smaller *f* by *f* matrix (see **Text S5: Recurrent implementation of CPA**), where *f* is the number of features of the input signal. Therefore, iCPA can handle very large dictionaries (**Fig. 5A**), as it does not invert a very large *n* by *n* matrix which is necessary for non-iterative CPA.

### CPA performance degrades with larger dictionaries and number of simultaneously present sources

CPA and iCPA assume a single dictionary element to represent each individual source present in an auditory scene. Therefore, the number of dictionary elements necessary in CPA is very high in order to represent all the sources that the system expects to encounter. Large dictionaries cause a deviation of CPA from its ideal behavior because larger dictionaries have more dictionary elements that are not present in the auditory scene. CPA uses a tiny bit of these spurious elements to generate its estimate of the observed signal 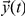, thereby reducing the contribution of the presence parameters of the elements that are actually present in the scene. This effect of the spurious elements increases with the total number of dictionary elements (**Fig. 5B**). Although diminished, the presence parameters of the elements that are part of the auditory scene are still much larger than the elements not present, allowing for perfect recognition. ICPA performance degrades as the number of sources present in a given auditory scene increases (**Fig. 5C**), since the multiple sources generate higher levels of overlap. Higher levels of overlap causes the scene to be more similar to other non-present dictionary elements therefore also reducing the presence parameters for the actual elements present. In order to handle larger dictionaries and more complex scenes, the auditory objects require representations with larger number of features (**Fig. 5D** **)** because as the number of features of the dictionary elements increases, the dictionary elements will be closer to being orthogonal and iCPA presence parameters will be closer to the ideal estimate, i.e. ones and zeros (**Fig. 5B**). As shown in **Text S4: Effects of auditory scene complexity and dictionary size on CPA performance**, the deviations from the ideal behavior for the presence parameters depends inversely on the square root of the number *f* of features.

### Iterative implementation of CPA is robust

**Figure 6:**
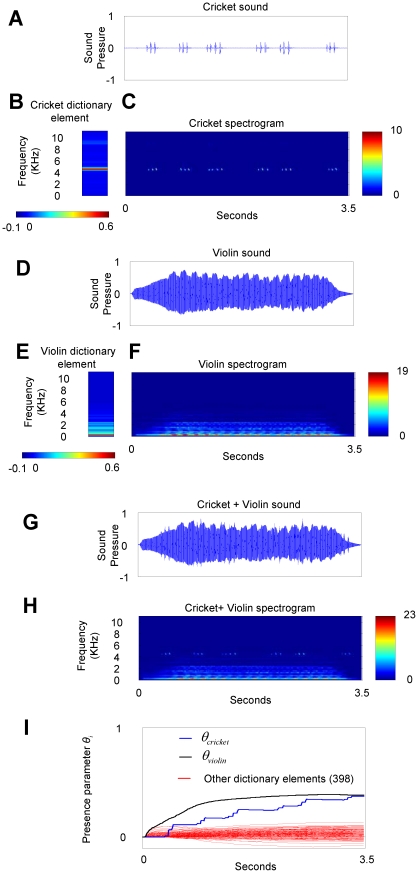
ICPA quickly identifies real auditory sources. Sounds produced by a cricket (**A–C**) and by a violin (**D–F**) were combined to create a complex scene (**G–H**). The dictionary elements that represented the violin and the cricket (**B** and **E**) were the average spectrum. (**I**) Using one second of data, iCPA calculated a presence parameter that was larger for the elements that represented the cricket and the violin. See also Audio S1, S2, S3.

The improved numerical robustness of iCPA permits the identification of real world sources, in which the description of a signal as a time varying spectrum is a good approximation (**Fig. 6A–F**). For the cases in which the spectrum is non-stationary, for example a source that consists of a frequency sweep, the simple feature space based on only the instantaneous spectrum would fail.

ICPA is also robust to the presence of unknown dictionary elements in an auditory scene because an element that is not part of the dictionary and, hence, is not represented by a large activity of a single presence parameter, shows up as a low activity profile that is spread across multiple dictionary elements (**Fig. S3**). This widespread low activity profile impairs the detection of the known elements if the new element is too loud (**Fig. S4**). The widespread low level activity profile could be used to indicate the presence of a new sound source that needs to be acquired [3].

### Auditory cortex has the connectivity to implement iCPA

The original formulation of CPA as an optimization problem is difficult to relate directly to a mechanistic model of brain processing. We therefore used iCPA to identify analogies to a dynamical model of a neuronal circuit. The operations necessary for iCPA can be implemented through synaptically connected networks of neurons. The iterative implementation of CPA (**Fig. 4**) receives as input the *f* variables of the signal 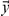 and expands the variables into the much larger number of variables 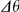 and ***K*** associated with the number *n* of dictionary vectors 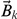. The variables 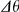 and ***K*** depend on the dictionary elements and they tend to be sparser than the input 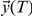.

ICPA also requires a massive feedback signal 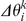 *i = 1,..,n*, *k = 1,..,f* to estimate the presence parameters 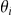. Both requirements comply with the characteristics of the auditory cortex since a) it expands the number of neurons associated with auditory representations and shows increased sparseness [8], [9], [10], [11], [12] compared to more peripheral areas [16] and b) sends massive corticothalamic projections [17] that could provide the feedback necessary for CPA. We thus hypothesize the primary auditory cortex to be the first place where neural activity represents the errors 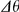 of the presence parameters as well as the associated uncertainties ***K***. Therefore, we analyzed differential behavior of ***K*** and 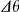 in order to understand how cortical neurons might represent these signals and to be able to identify such units from physiological recordings. The variables ***K*** and 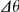 were decomposed into single components that could be mapped into cortical neuron activity. Although there are multiples ways to represent a matrix, we choose a representation that assigned to each of these “neurons” a preferred frequency, corresponding to the features of the vectors of the dictionary elements (see **Identification of elements in the model as cortical cell activity** for more details).

### The parameter error 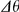 behaves differently from the uncertainty parameter *K*

The elements of ***K*** should have a large value if the input 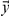 is low for a period of time because, when there is not enough accumulated information about which sources are present, the algorithm should adjust the parameters 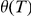 by a large margin, as the estimated presence parameters are likely to be different from the actual presence parameters. As more samples of input 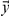 are collected, the estimated presence parameters 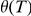 will better match the real presence parameters, and they should not require much adjustment. This should be reflected in smaller values for the elements of ***K***. Therefore, the behavior of ***K*** matches the time course of the uncertainty about which sources are present; in the silence preceding a scene there is a large uncertainty about which sources are present. As the scene continues, there is more information and the uncertainty diminishes. We therefore labeled the elements of ***K*** the uncertainty associated elements.

**Figure 7:**
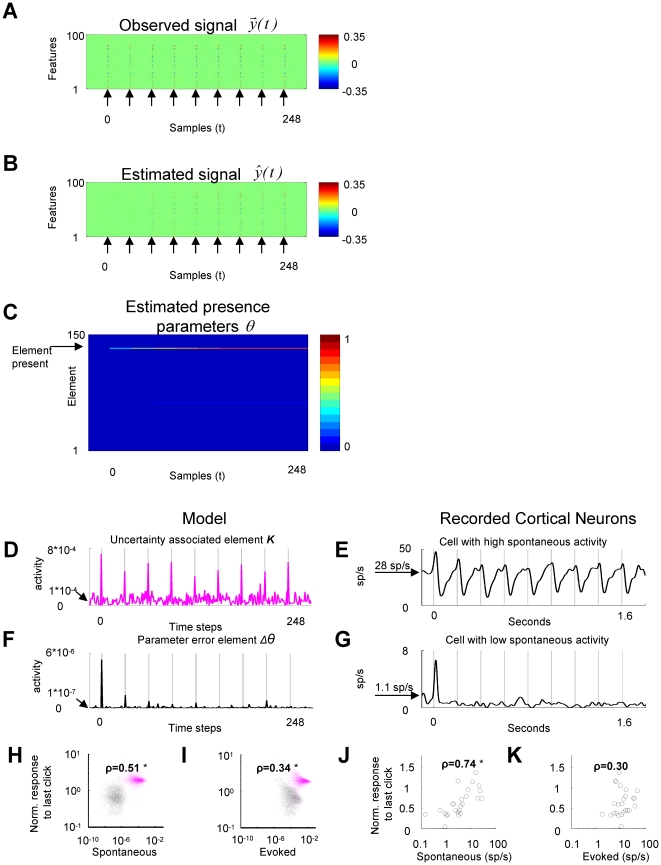
ICPA behavior of uncertainty encoding elements and error encoding elements matches the behavior of cortical neurons. (**A–C**) **Simulation of iCPA in response to a train of clicks.** (**A**) The click was simulated by a single dictionary element that was presented periodically as indicated by the arrows. (**B**) The observed signal 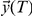 was initially different from the estimate 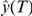. When the train of clicks continued, the estimated signal approximates the observed signal. (**C**) A (single) correct dictionary element is identified. (**D–F–H–I**) (**D**) Example of an uncertainty encoding element of ***K*** *(T)* showing its higher spontaneous activity and lesser adaptation in response to the click than (**F**) the example of an error encoding element of 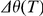. (**H**) Spontaneous activity (before click train) and adaptation in response to the click are correlated when the ***K*** *(T)* (magenta) and the 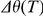 elements (black) are grouped together. (**I**) The correlation between the evoked activity (response to the first click) and the adaptation was weaker. (**E–G–J–K**) Activity recorded in the awake rat auditory cortex in response to a 5 click/sec train shows similar relationships between spontaneous activity and adaptation. Similar results occurred in response to a 20 click/sec train (**Fig. S5**) and after subtracting the spontaneous activity from the evoked responses (**Fig. S6**). The asterisk indicates a Spearman rank correlation with significance p<0.01.

The error in the presence parameter 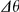 depends not only on ***K*** but also on the estimation error 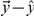 (**equation 5**), which causes a difference in behavior between 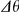 and ***K***. In order to illustrate this behavior, we have simulated the responses of a train of clicks (**Fig. 7A**), each click consisting of a single dictionary element. The train of pulses was preceded by a brief period of silence. During the silent period preceding the train, the estimated signal 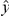 (**Fig. 7B**) is close to zero. Therefore, the estimation error 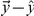 has a very low value in the absence of input as both the input 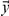 and the estimate 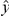 are close to zero. The estimation error 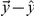 is very high as the click train starts and again decreases as the estimate 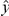 becomes a better match to the input signal 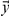 (**Fig. 7B**). The estimation error 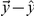 decays as the correct single presence parameter is estimated (**Fig. 7C**).

The error in the presence parameter 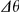 is calculated by multiplying the uncertainty ***K*** by the estimation error 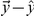. The low level of activity of 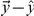 preceding the start of the stimulus makes the presence parameters 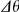 less spontaneously active than the uncertainty ***K***. The effect of the decay of 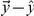 also causes the presence parameters 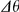 to decay even more than uncertainty ***K***. These two effects are illustrated by the simulation results in **Fig. 7D–F–H**. In the absence of input, the elements of the uncertainty ***K*** (**Fig. 7D**) have larger activity than the error in the presence parameter 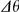 (**Fig. 7F**). The evoked activity decreases for both types of signals as the train of clicks continues, but with the error in the presence parameter 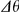 decaying more strongly than the uncertainty ***K***. The estimation error 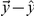 hence causes the parameter error elements 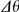 to have less activity than the uncertainty elements ***K*** both in the absence of input and while the auditory scene is not changing (**Fig. 7H**).

ICPA makes a prediction about the differential behavior of neurons representing the parameter error 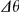 and sensitivity ***K*** in the absence and presence of sensory information. We therefore asked if the auditory cortex would show cells with activity similar to 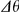 that depended on the error estimate 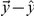. The effect of the error estimate would cause these cells to show less response both in the absence of sound and during repetitive sounds than nearby cells that would represent the uncertainty associated variables ***K***. Although other models [4], [18], [19] as well state that the cortex represent error estimates 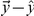, iCPA furthermore predicts the existence of the two distinct response populations.

### Auditory cortical response matches the behavior of the 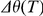 and *K(T)*

As a first step to test for the hypothesis that the thalamocortical circuit implements iCPA, we reanalyzed single units from the awake rat auditory cortex [10] in response to a train of clicks. In the absence of sensory stimulus, different cortical cells show different levels of spontaneous activity [9], [20], [21] and different levels of activity in response to a repetitive sound [22]. In agreement with the prediction of iCPA, cells with high spontaneous firing rates (**Fig. 7E**) adapted less than cells with lower spontaneous firing rates (**Fig. 7G** and **Fig. 7J** for population data), indicative of the multiplicative effect of the error estimate 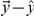. A simple model in which a cell's spiking threshold determines both the spontaneous activity and the degree of adaptation would also produce a strong correlation between the evoked responses and adaptation. However, both the model (**Fig. 7I**) and the neural recordings (**Fig. 7K**) exhibit weaker correlations between the evoked responses and adaptation. ICPA provides a computational explanation for this correlation between two seemingly unrelated features of activity in primary auditory cortex.

This data does not indicate if the elements 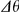 are represented by different populations of cells than the uncertainty associated elements ***K***, or if there is a continuum in how strongly the estimation error modulates a cell's activity. By recording from identified neural populations, it will be possible to test if the parameter error elements 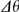 and uncertainty associated elements ***K*** are represented by distinct neural populations.

## Discussion

We proposed a new algorithm, called CPA, which identifies the sources present in a complex auditory scene. CPA belongs to a family of algorithms that identify the few elements from a large dictionary of possible sources that are used to reconstruct the signal. CPA differs from similar algorithms in that the estimated parameters indicate only the presence or absence of the corresponding dictionary element in the mixture and are independent of the magnitude of the contribution of the dictionary elements to a particular scene. The parameters do not change on the fast time scale of sensory input fluctuations and match the psychophysics of auditory stream perception. We have shown that CPA can be implemented as an iterative estimator, in which the current estimate about which sources are present is corrected depending on the mismatch between a new sensory observation and an estimate on what the scene should be. The iterative CPA predicts that the expanded cortical representation should show responses that represent the error in the presence parameters and others that signal the uncertainty about the presence parameters. Cortical recordings of awake behaving rats included both response types predicted by the model.

### Model limitations

CPA implies that a single auditory source is represented by a single dictionary element, which is in contrast to other sparse representation approaches where a single source can be represented by more than one dictionary element [1], [2], [3], [4], [5], [6]. Therefore, auditory scenes in CPA are represented extremely sparse (for an example see Fig. 5A, where 2 elements out of 68000 are active), which seems at odds with the lower levels of sparseness in auditory cortex, although high levels of sparseness have been reported [16]. Below (section **Sparse activity in the auditory cortex**) we argue how this problem could be resolved.

In order to identify a source, CPA also requires that each single element that corresponds to a source should already be part of the dictionary. If such element is not yet part of the dictionary, CPA will, of course, fail to recognize this sound in that the source won't be assigned a single presence parameter. However, an unknown source evokes small values over multiple presence parameters (**Fig. S3**) providing an indication that something unfamiliar is being presented which could be added to the dictionary. In order to create the dictionary, the animal should be continuously acquiring the sources that it is exposed to. Although there are multiple algorithms that are capable of learning these sparse overcomplete representations [3], [5], [23], it is not clear what algorithm is used by the brain to create the dictionary.

Concerning the implementation of iCPA, we have argued in favor of the hypothesis that the auditory cortex is the place where the conversion from a signal estimation error into a presence parameter error occurs. We base this hypothesis on the sparseness of the response and the massive corticothalamic feedback. Also, other implications of iCPA for activity signaling errors in the presence parameters seem to coincide with reported features of auditory cortex (see sections **Two types of neuronal responses in auditory cortex** and **Cortical activity as estimation error**), and these features have not been reported for other brain areas. However, this all is far from being a proof that the algorithm is implemented in the corticothalamic system. Specifically feedback is a quite general feature across different levels of the auditory system, including inferior colliculus [24]. Also, other non-primary areas have very selective responses to sounds [25].

Finally, the model cannot yet deal with non-stationary auditory scenes in which sources dynamically appear and disappear on a slow time scale. However, an extension to such a situation could be implemented rather easily by introducing a slow temporal decay of the presence parameters.

### Sparse activity in auditory cortex

Although responses in auditory cortex are sparse [8], [9], [10], [11], [12], meaning that only a minority of cells respond to any particular stimulus, the levels of sparseness mostly observed in auditory cortical recordings are not as extreme as we would expect from a representation of CPA's presence parameters. There are two reasons for this apparent discrepancy between the cortical recordings and the predictions from iCPA. The first one is that in iCPA the sparseness is maximal for the presence parameters 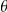. However, the uncertainty of the parameters ***K*** and the error in the parameters 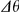, which we hypothesize to be represented in cortex, are less sparse, because they are driven by the estimation error 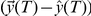 and the projections 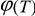, which are non-sparse signals. Nevertheless, very particular subpopulations (L3) of neurons in auditory cortex do show very high levels of sparseness, compatible with representations of presence parameters(see **Fig. 6** in [26]). Secondly, the particular level of sparseness measured on the response of a single neuron involved in the representation of presence parameters depends on the particular neural code that is used. If a particular presence parameters is represented by the activity of a subset of neurons, each single cell could be part of multiple of such subsets, with the activity of each neural ensemble representing a single presence parameter [27], [28]. Simultaneous recordings of large a number of neurons would be needed to identify the particular neural population code used to represent the presence parameters [11], [12]. CPA predicts that the presence parameter activity will be sparser for auditory sources recognized by the animal as opposed to new sources, because a new source is represented by multiple small presence parameters and not by a single large one (see **Fig. S3, S4**).

### Cortical activity as estimation error

The iterative implementation of CPA, as well as other frameworks of cortical function [4], [18], [19] propose that cortical activity encodes the difference between the sensory signal and an estimate of that signal. This estimate is calculated using an internal model of the world. When this internal model approximates the external world well, the estimate will be a close match to the incoming signal. A system that is actively refining its internal model would show a paradoxical reduction of cortical activity, compared to a system where the internal model is not being refined. Consistent with this theoretical framework, the auditory cortex evoked activity is reduced during auditory discrimination tasks, where the animal might be improving its internal model, compared to passive hearing conditions where this improvement is not required [10], [29], [30]. Moreover, our model predicts that the reduction should be confined to the cells that represent the error in the parameters, as they receive as input the difference between the sensory signal and an estimate of that signal (**Fig. 4**). This contrasts with cells that represent the uncertainty that do not receive such input, and should not show such reduction. We postulate that the parameter error elements can be identified by their low spontaneous activity. In fact, suppression of evoked activity during behavior was confined to low spontaneously active cells, matching the prediction from iCPA (see Supplementary Figure S4 in [10]).

### Two types of neuronal responses in auditory cortex

ICPA makes a prediction about two types of behaviors in cortical cells, with one type encoding the error in the presence parameters and a second type encoding the associated uncertainty. We postulate that these two populations could be distinguished by their levels of spontaneous activity and we found that the spontaneous activity can be used to determine the level of adaption, which according to iCPA differs for the two cell populations. There are two pairs of known candidates. One pair consists of the fast spiking interneurons and the regular spiking neurons in which fast spiking interneurons have higher firing rates than regular spiking neurons [20]. The other pair are lower layer cells and upper layer cells in which lower layer cells have higher spontaneous firing rates than upper layer cells [9], [21]. According to iCPA, the high spontaneous cells drive the behavior of the low spontaneous ones. Therefore, the high spontaneous population should have shorter sound evoked latencies than the low spontaneous ones. In fact, the fast spiking neurons have been reported to have shorter latencies than regular spiking neurons [31] and lower layer neurons also have shorter latencies than upper layer neurons [21]. Selective recordings of these populations of neurons during the performance of sound identification tasks in complex scenes might narrow down the possible populations that are involved in representing uncertainty and the error in the parameters.

### Role of corticothalamic feedback

The proposed model provides a mechanism for how the corticothalamic system solves the source identification problem in agreement with the observed physiology of stream segregation in auditory cortex [32], [33]. The model suggests that source identification in complex scenes combined with attention-modulated auditory cortical activity [34] allows to selectively attend to a source when multiple sources are simultaneously present, solving the cocktail party problem. Furthermore, we provide a computational hypothesis for the massive feedback connections in the corticothalamic loop. This feedback is a fundamental property of the proposed circuit. Our model, in fact, predicts that blocking corticothalamic feedback would impair the capability to identify a source in complex auditory scenes. We propose that these connections convey error signals about which few out of the large number of dictionary elements are present. Therefore, this error signal should show fast adaptation, as the correct presence parameters are estimated. Other models consider that the corticothalamic feedback represents the estimate about the observed signal [3], [4], [19]. In those models, the feedback signal should show no adaptation.

### Features used to characterize auditory sources

We have used the spectrogram to characterize the auditory sources [13] and showed that it was sufficient to identify some natural sounds. The spectral structure is a powerful feature for the segmentation of natural sounds [35]; large spectral overlap impairs the separation of sources in humans [36]. Cortical neurons show tuning to complex spectrotemporal features [37] which could be included as extra features for the dictionary element. Beyond spectral and spectrotemporal features, source separation is also supported by spatial cues [38], which we have not included in the present approach. This extra information can be incorporated into the current framework by generalizing a dictionary element into several positions [2].

### Experimental evidence of new source acquisition

In order to be able to identify sources in a complex auditory scene, CPA requires that all the sources present are already stored as dictionary elements. Therefore, the auditory system should be learning samples of all the sounds that it encounters to improve its performance of sound identification in complex scenes. Although we do not know how extended this learning of sound is, a recent study has shown that humans, without being aware, learn a random spectro-temporal modulations of noise [39] with only a few presentations and retain that information for several weeks. This is consistent with the idea that the auditory system is continuously acquiring new sounds and incorporating them to a dictionary. A prediction from the CPA algorithm is that masking by an unknown sound should be more effective than masking by known sounds.

### Presence parameters as auditory streams?

We note that the presence parameters 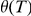 do not reflect the fluctuating contribution of a particular dictionary element to the auditory scene, but are calculated considering all previous observations. The presence parameters are updated after each new observation and the value corresponding to an element present starts growing at the onset of the auditory scene. The estimated presence parameters converge to a constant value although the amplitudes of the sources fluctuate in time. The presence parameters keep their values, even if their contributions temporarily fall to zero (**Fig. 6**). In this sense, the slow buildup and continuity of the presence parameters matches the psychophysics of auditory streams [35], more than the quickly fluctuating parameters estimated by other algorithms (**Fig. 1E–F**) [1], [2], [3], [4], [6]. We thus may interpret the “active” presence parameters as auditory streams. Although it is possible to calculate a stable presence parameter based on the fluctuating parameters calculated by other algorithms (see equation 2), these derived quantities are not used by those algorithm. In the case of iCPA, the presence parameters are essential for the functioning of the algorithm and appear on the feedback loop. One prediction of iCPA is that the corticothalamic feedback elements' responses should be amplitude invariant and reflect the psychophysics of auditory streams.

### CPA for other modalities?

Analyzing scenes composed of amplitude modulated sources is a problem that also appears in other modalities, such as olfaction [14]. The olfactory bulb also receives a large feedback signal from the piriform cortex, another large, sparsely active structure [40], suggesting that a similar algorithm might be implemented already in paleocortex to perform olfactory source identification in natural scenes.

## Materials and Methods

### Corrected projections algorithm (CPA)

The estimation of the presence parameters in CPA was done by finding the set of presence parameters 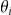, *i = 1..n* that will minimize the average minimal square error between the signal 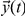, *t = 1..T*, and the estimate of that signal 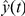. The estimate 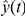 is given by the linear combination of all the projections of the signal 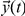 onto each and all of the dictionary elements 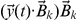, *k = 1,..,n*,
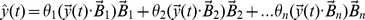
The dictionary elements 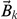, *k = 1,..,n*, are unit vectors. This problem can be solved as linear least square minimization [41]. By arranging the observations and the projections as matrices, it is equivalent to an inversion of *n by n* matrix.

The auditory scene consisted of 180 samples of a 10 dimensional mixture signal generated by the linear combination of two vectors, given by:
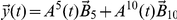
For visualization purposes, the elements of the two vectors 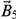 and 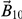 originated from a lognormal distribution of mean zero and variance 1. Afterwards, the vectors had their mean subtracted and were normalized to unit length. The temporal modulations 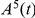 and 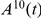 were generated by independent normal variables of zero mean and unit variance.

The dictionary consisted of additional 16 vectors, whose elements were taken from a normal distribution of mean zero and variance one. All the 18 dictionary elements were normalized to have unit value and zero mean.

The dictionary of elements used was the same as **Figure 3 A**. The standard deviation of the temporal modulation 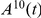 associated with element 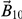 was reduced to 0.1, while the standard deviation of 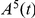 was kept at one.

### Iterative Corrected projections algorithm (iCPA)

ICPA consists of calculating a new *n*-dimensional presence parameter column vector 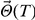, based on the previous presence parameters and a new observation of the auditory scene, expressed as an *f*-dimensional column vector 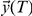. This observation 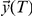 is projected onto the *n* dictionary elements, creating an *f by n* matrix 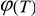 with elements
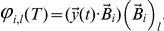
Each column of 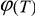 is hence given by the projection of the observation onto each dictionary element. Then the estimate of the observed signal is computed as
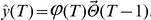
The new presence parameter is determined as
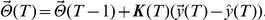
Note that the update of the presence parameter depends on the *n by f* matrix
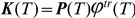
in which the *n by n* matrix 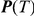 is obtained from the iterative equation
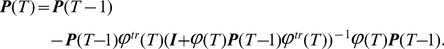
The matrix transpose is indicated by the ^tr^ symbol.

As an initial value for 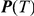 we use a diagonal matrix, that is most of the elements of matrix are initially zero. The matrix 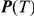 acts as memory of uncertainty about sources present in a scene. **Figures 5**, **6**, and **7A–B–C** were generated employing the above equations.

We evaluated iCPA with T = 10 observations of the auditory scene. There, the estimated presence parameters reached a steady state. The components of dictionary elements were taken from a uniform distribution between zero and one. Afterwards, each dictionary element had its mean subtracted and were normalized to unit length. The auditory scenes were generated by taking a few elements from the dictionary and modulating them using a normally distributed amplitude modulation with zero mean and variance 1. The matrix 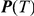 was initialized as the identity matrix.

The sound produced by the cricket and the violin, playing a C4 were sampled at a frequency of 22050 Hz. In order to generate the spectrograms, a window of 10 ms was used, yielding 111 frequency bands. This would correspond to *f = 111* features for the dictionary elements. The spectrograms were calculated using the Matlab function SPECGRAM with each window smoothed using a Hanning window.

The dictionary elements that represent the violin and the cricket were calculated by taking the average spectrum of the presentation of the cricket and the violin. These average spectra were incorporated as two of the 400 dictionary elements.

In order to generate the other 398 dictionary elements, a collection of music files were resampled to a frequency of 22050 Hz and the spectrograms were calculated using the same parameters as the violin and the cricket sound. Spectrograms were calculated for 30 seconds segments and the Principal Component that captured 95% of the variance of the spectrogram was incorporated into the dictionary, yielding a dictionary with elements that matched the spectra of natural sounds. The matrix 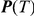 was initialized to a diagonal matrix of value 5e-4.

We used a signal of *f = 100* features and a dictionary of 150 elements. Each element was a *100*-dimensional column vector. The elements of dictionary elements were taken from a uniform distribution between zero and one. Afterwards, each dictionary element had its mean subtracted and it was normalized to unit length. The observed signal 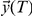 was a *100*-dimensional column vector and consisted of a train of 9 pulses. The pulses were generated by applying one of the elements of the dictionary with unit amplitude for one time step. The silence period between pulses was equal to 30 time steps. Constant additive Gaussian noise with zero mean and standard deviation of 0.002 was added to the pulse train. The simulation initial parameters 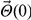 were random small values, with mean 0.012 and standard deviation 0.006. The initial value for the ***P*** *(0)* matrix was a diagonal matrix with the diagonal elements equal to 0.5.

### Identification of elements in the model as cortical cell activity

We assumed the cortex to receive two types of inputs. The first one is the error in the estimation 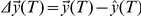 in which the estimate 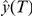 was calculated in the thalamus, by way of the massive corticothalamic feedback connections. The second input is the current observation 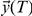. This current observation can be converted through appropriate synaptic weights into the projection onto all the dictionary elements, expressed as 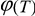.

The other elements required for the implementation of iCPA are the matrices 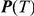 and 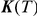, and the vector 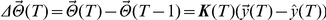. In response to a single stimulus, only a few elements on the diagonal of the matrix 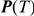 are active. Therefore, although 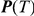 has many elements, we do not expect that its activity would be reflected in responsive cortical cells.

Cortical cells have a preferred frequency [42]. Therefore, in order to be able to identify the cortical activity as the elements of 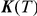 and 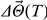, we decomposed them into frequency bands. In the case of the *n* by *f* matrix 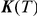, the simplest decomposition is to consider each of its elements to represent cortical activity, with each element having a best frequency corresponding to its column number, that is each 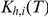, *h = 1..n*, *i = 1..f* is considered as the response of one particular neuron or group of neurons.

In the case of the *n* dimensional parameter error signals 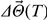, the decomposition into elements associated with the *f* frequencies is not one-to-one. However, we can naturally decompose the error signal 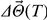 vector into elements that have a frequency preference by using the matrix multiplication
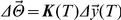
where
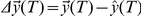
This matrix multiplication represents the following equations:
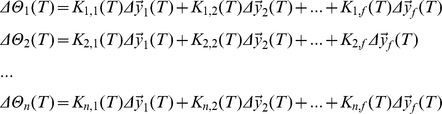
We identified the terms in the sum 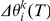 = 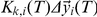 as being represented by cortical neurons because these terms have a preferred frequency. Although spiking activity can be only positive, 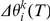 and 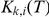 can have both positive and negative values. To map these numbers to firing rates, we have taken their absolute value, which assumes different cell populations to encode for the positive and the negative values.

Both groups of elements, 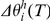 and 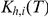, *h = 1∶150*, *i = 1∶100*, were combined together to calculate the correlations. The Spearman correlation between the spontaneous activity and the normalized response of the last pulse was r = 0.51, p<1e-12, with the 95% confidence interval between 0.50 and 0.52. The correlation between the evoked activity and the normalized response of the last pulse was smaller, r = 0.34, p<1e-12, with the 95% confidence interval being between 0.33 and 0.35.

### Auditory cortical recordings

We used previously published data of 32 awake rat auditory cortex neurons in response to trains of clicks. For more details, see [10]. We analyzed the responses to a train of 5 clicks per second, 1.8 seconds (9 clicks). Spontaneous activity was evaluated in a 20 ms window preceding the onset of the first click. Evoked activity in response to the clicks was evaluated in a 20 ms window between 6 and 26 ms from the onset of each click. To quantify the accommodation, the evoked responses to the train of clicks were normalized to the response to the first click and fitted to the decaying exponential:
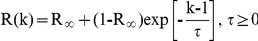
using the Matlab function LSQCURVEFIT. We used the estimated parameters to each cell to estimate the responses after 9 pulses of the 5 clicks per second train. Out of the 32 cells, 25 cells could be fitted with less than 10% error with the decaying exponential function.

The correlation coefficients were calculated using the Spearman's rank correlation coefficient. The correlation between the spontaneous activity and the normalized response of the last pulse was r = 0.74, p = 2.81e-5, with the 95% confidence interval of the correlation coefficient between 0.48 and 0.88. There was no significant correlation between the evoked response to the first click of the train and the normalized response of the last pulse (r = 0.30, p = 0.14, with the 95% confidence interval of the correlation coefficient between −0.11 and 0.62).

## Supporting Information

Figure S1 **Sources that have some spectral overlap are still separable using CPA.** (**A**) Two elements that show some degree of overlap are mixed. The figure has the same structure as **Fig. 3A–F**.
(TIF)

Figure S2
**ICPA can identify sources present with less observations of the auditory scene than Principal Component Analysis.** We would like to compare the capability of iCPA in identifying the sources that generated a signal with the capability of Principal Component Analysis (PCA) as a function of the number of observations of the auditory scene. We generated an auditory scene by using 10 vectors of f = 500 features, selected from a dictionary of n = 1000 possible elements, amplitude-modulating them and combining the amplitude modulated signals. The amplitude modulation of each source, at each time step, is given by is a number, uncorrelated across the 10 sources and uncorrelated in time, taken from a lognormal distribution of log mean value of zero and log standard deviation of 2. At each time step, the combination of the 10 signals created the auditory scene. Besides, at each time step, a 500 dimensional uncorrelated noise, taken from a Gaussian distribution with zero mean and 0.5 standard deviation, was added to the auditory scene. We evaluated iCPA and the Principal Component based method by assessing its performance in identifying the sources present for different number of observations of the signal. The performance of iCPA was given by how many of the 10 largest 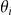 identified corresponded to the actual dictionary elements involved in generating the signal. Standard PCA, on the other hand, identifies the elements based only on the observed auditory scene and does not use the information that the elements that generated the signal are taken from the dictionary of 1000 possible elements. In order to be able to obtain a performance index for PCA similar to the one that we calculated for iCPA, we first calculated the principal components. We took the 10 largest principal components and for each principal component, we identified the element from the dictionary that was the better match to that identified principal component. The performance of PCA was given by how many of the 10 best matches corresponded to the actual dictionary elements that generated the signal. We repeated the procedure 30 times for each number of observations. The shadings indicate ± the standard deviation calculated for the 30 repetitions. The figure shows that iCPA (in red) required 9 observations to reach a performance of 80%. IPCA (in blue) required 66 observations to reach similar performance.(TIF)

Figure S3 **Estimated parameters for a source that is not part of the dictionary is distributed across multiple dictionary elements.** ICPA was presented with a source of f = 300 features that was not part of a dictionary of n = 1000 possible sources, with an amplitude of 1. The new element appears as low level activation on multiple presence parameters.
(TIF)

Figure S4 **CPA can identify dictionary elements even in the presence of unknown elements.** (**A**) ICPA identifies the two random non-orthogonal sources of f = 300 features using a dictionary of n = 1000 possible sources. The mean amplitude of these known dictionary elements was one. (**B–D**) Adding an extra source that is not part of the dictionary with increasing standard deviation amplitude of 0.25, 0.5 and 1 causes the larger level of background activation in the presence parameters. However, the iCPA is robust to the presence of this “non-dictionary” element.
(TIF)

Figure S5 **There was a correlation between spontaneous activity and normalized response of the last click for a 20 click per second train.** (**A**) There is a significant correlation between the spontaneous firing rate and the normalized response of the last click of a 20 click/sec (**B**) There was no significant correlation between the normalized response of the last pulse and the response evoked by the first click (see **Text S6: Statistics**).
(TIF)

Figure S6 **Correlations between normalized response of the last click and spontaneous activity was maintained after subtracting the spontaneous activity.** There was a significant correlation between the spontaneous activity and the normalized response of the last click of (**A**) the 5 click/sec train and (**C**) the 20 clicks/sec train. The responses to the clicks were calculated by subtracting the spontaneous activity from the evoked response. There was no significant correlation between the spontaneous subtracted normalized response of the last click and the evoked activity for neither the 5 clicks/sec train (**B**) nor for the 20 clicks/sec train (**D**). (See **Text S6: Statistics.**)
(TIF)

Text S1 **Definition of auditory scene.**
(DOC)

Text S2 **Corrected projections algorithm.**
(DOC)

Text S3 **Proof that CPA detects the elements present in a mixture.**
(DOC)

Text S4 **Effects of auditory scene complexity and dictionary size on CPA performance.**
(DOC)

Text S5 **Recurrent implementation of CPA.**
(DOC)

Text S6 **Statistics.**
(DOC)

Audio S1 **Cricket only.**
(WAV)

Audio S2 **Violin only.**
(WAV)

Audio S3 **Cricket+violin.**
(WAV)
