# Supplementary figures and images for: A Corticothalamic Circuit Model for Sound Identification in Complex Scenes
Source: PLoS One. 2011 Sep 13;6(9):e24270. doi: 10.1371/journal.pone.0024270 (PMC3172241; doi:10.1371/journal.pone.0024270)

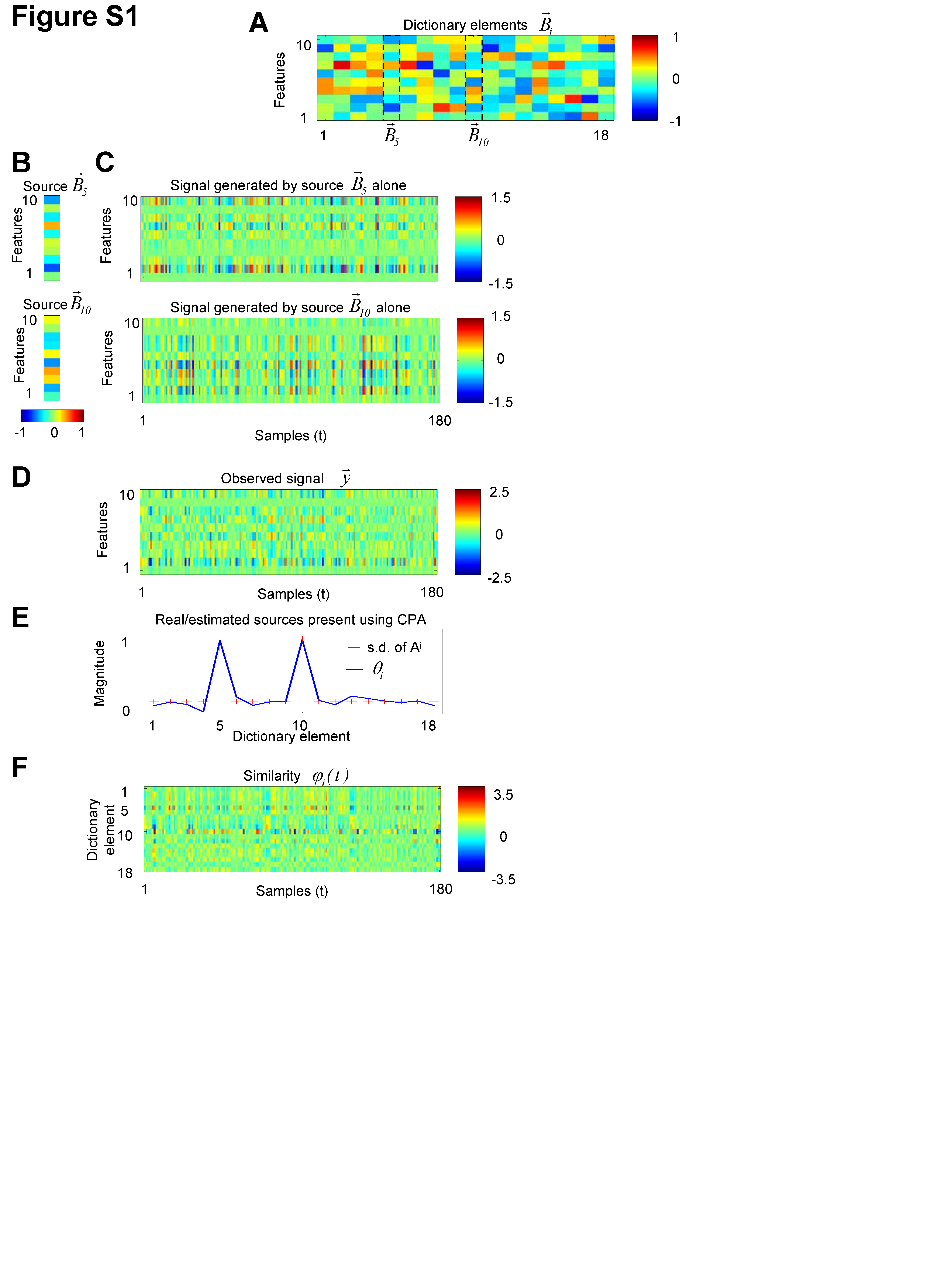

Supplement: Figure S1 — Sources that have some spectral overlap are still separable using CPA. (A) Two elements that show some degree of overlap are mixed. The figure has the same structure as Fig. 3A–F . (TIF) [file pone.0024270.s001.tif]

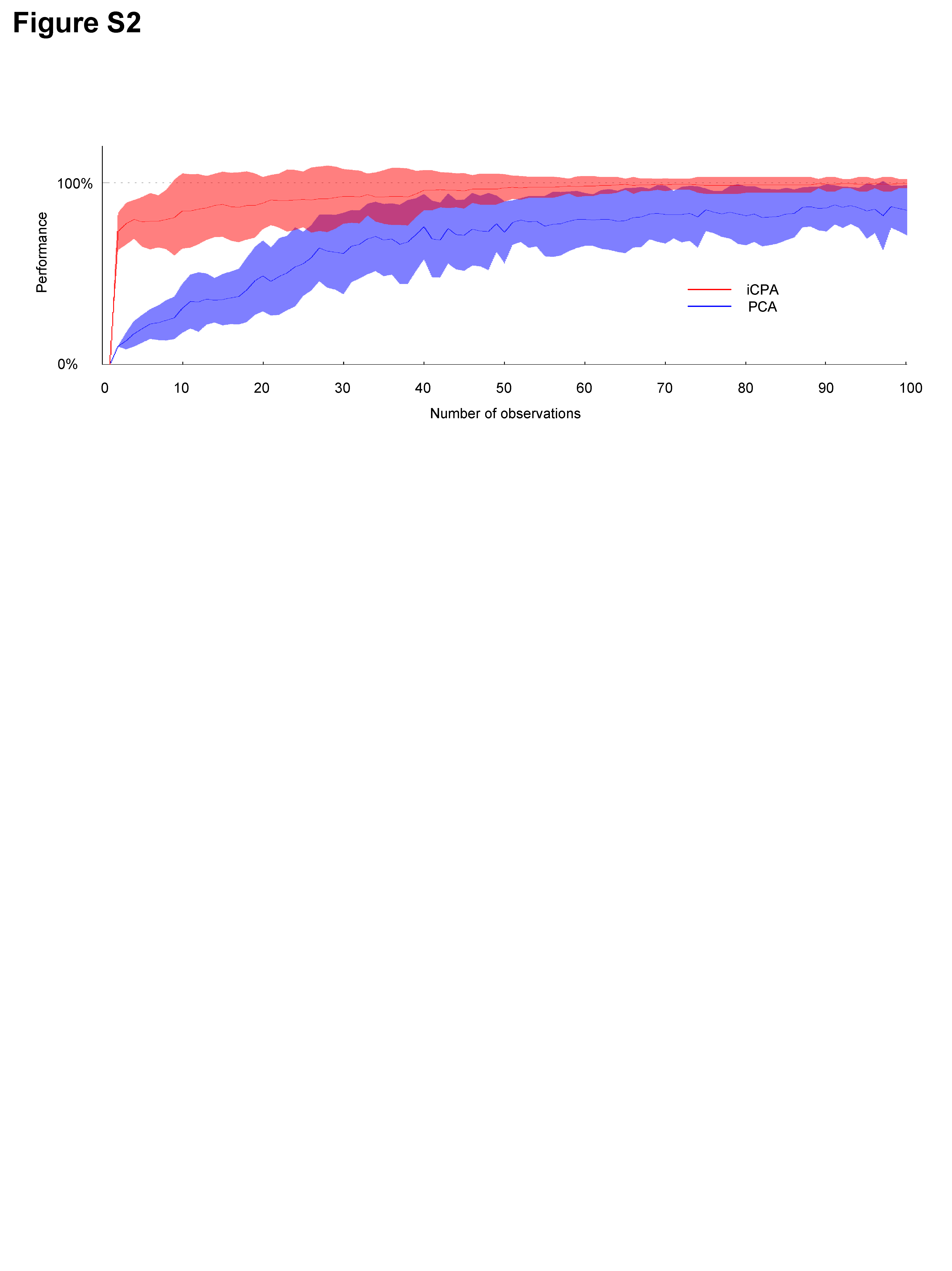

Supplement: Figure S2 — ICPA can identify sources present with less observations of the auditory scene than Principal Component Analysis. We would like to compare the capability of iCPA in identifying the sources that generated a signal with the capability of Principal Component Analysis (PCA) as a function of the number of observations of the auditory scene. We generated an auditory scene by using 10 vectors of f = 500 features, selected from a dictionary of n = 1000 possible elements, amplitude-modulating them and combining the amplitude modulated signals. The amplitude modulation of each source, at each time step, is given by is a number, uncorrelated across the 10 sources and uncorrelated in time, taken from a lognormal distribution of log mean value of zero and log standard deviation of 2. At each time step, the combination of the 10 signals created the auditory scene. Besides, at each time step, a 500 dimensional uncorrelated noise, taken from a Gaussian distribution with zero mean and 0.5 standard deviation, was added to the auditory scene. We evaluated iCPA and the Principal Component based method by assessing its performance in identifying the sources present for different number of observations of the signal. The performance of iCPA was given by how many of the 10 largest identified corresponded to the actual dictionary elements involved in generating the signal. Standard PCA, on the other hand, identifies the elements based only on the observed auditory scene and does not use the information that the elements that generated the signal are taken from the dictionary of 1000 possible elements. In order to be able to obtain a performance index for PCA similar to the one that we calculated for iCPA, we first calculated the principal components. We took the 10 largest principal components and for each principal component, we identified the element from the dictionary that was the better match to that identified principal component. The performance of PCA was given by how many of the 1 [file pone.0024270.s002.tif]

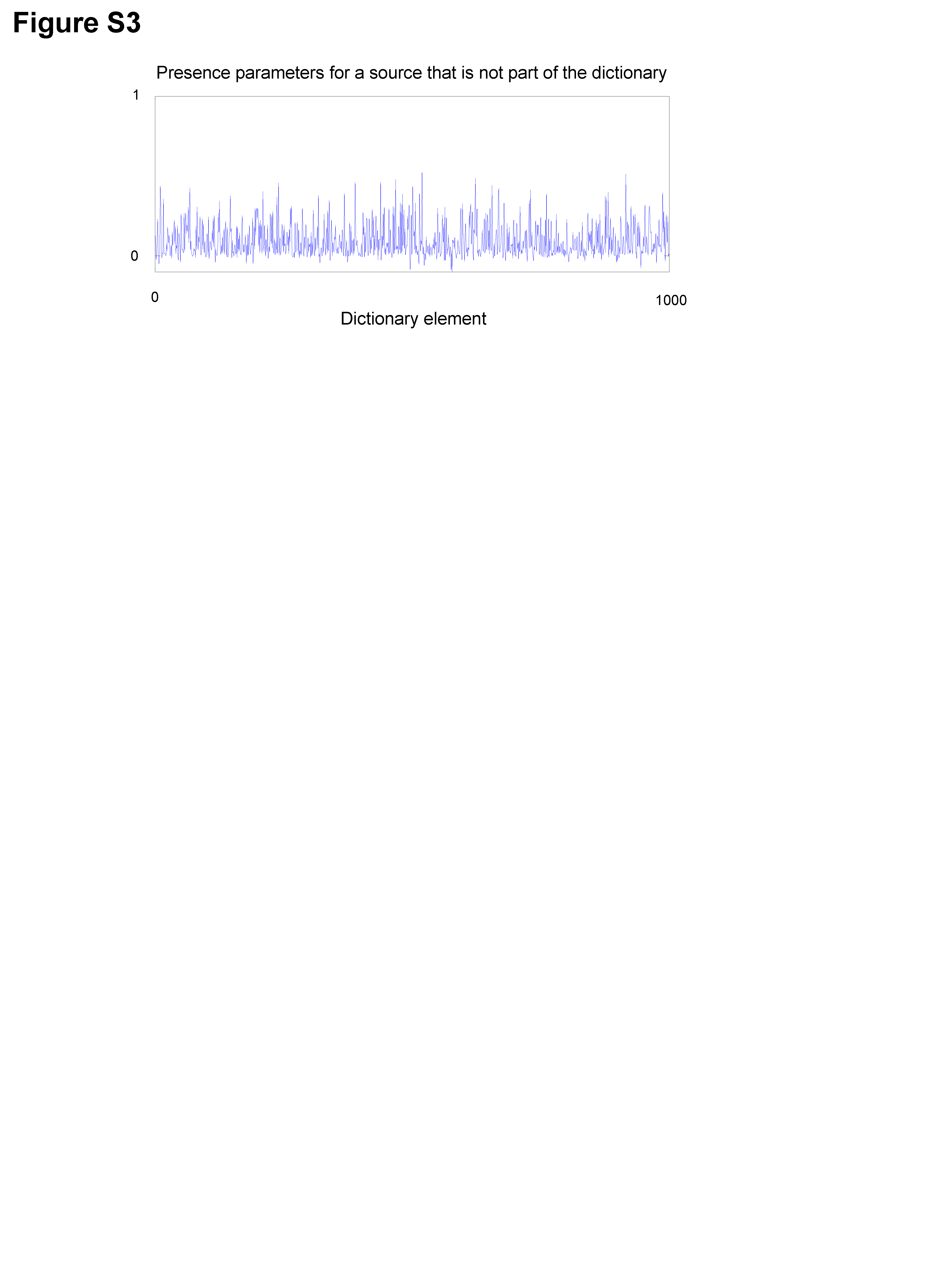

Supplement: Figure S3 — Estimated parameters for a source that is not part of the dictionary is distributed across multiple dictionary elements. ICPA was presented with a source of f = 300 features that was not part of a dictionary of n = 1000 possible sources, with an amplitude of 1. The new element appears as low level activation on multiple presence parameters. (TIF) [file pone.0024270.s003.tif]

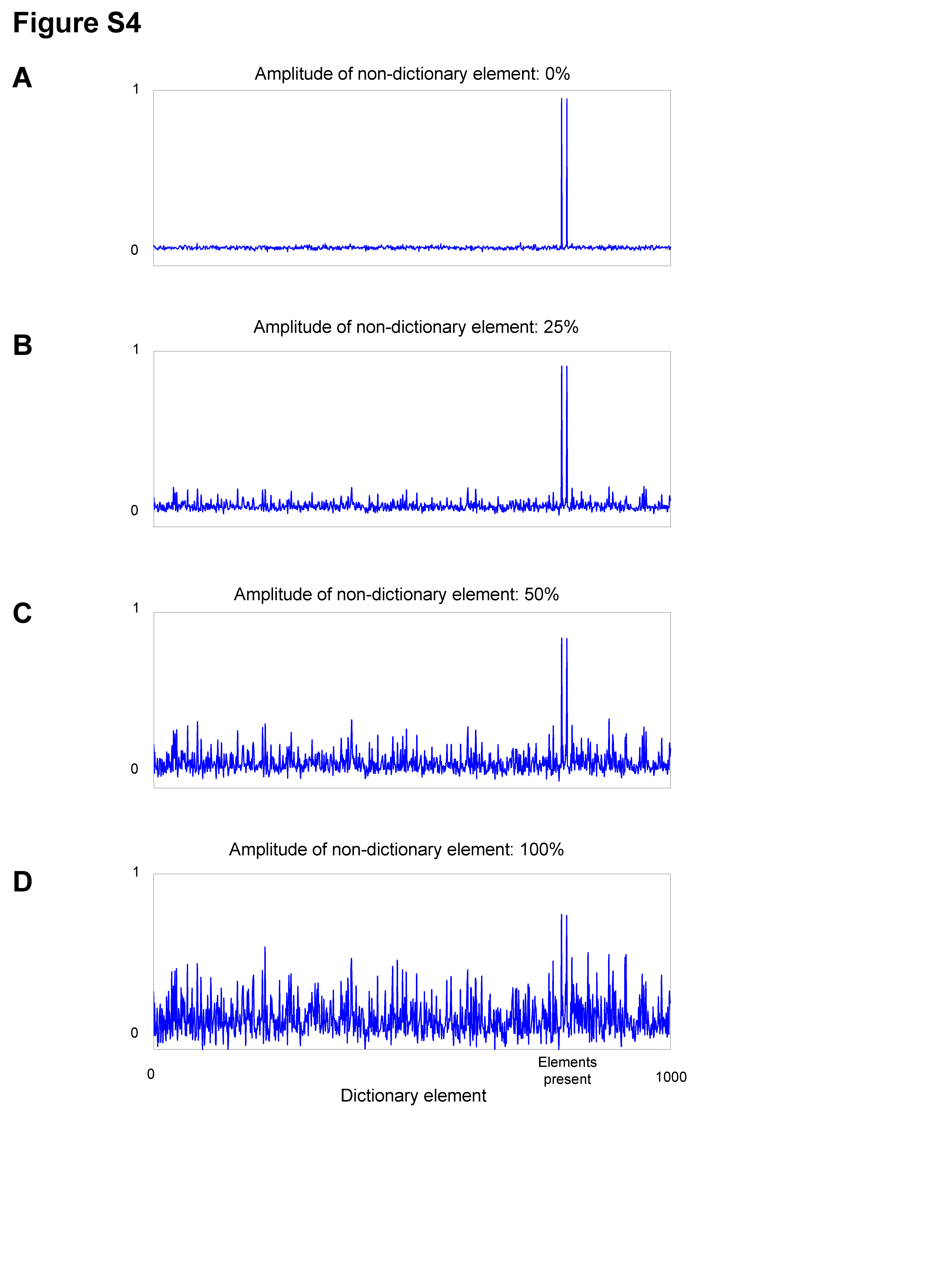

Supplement: Figure S4 — CPA can identify dictionary elements even in the presence of unknown elements. (A) ICPA identifies the two random non-orthogonal sources of f = 300 features using a dictionary of n = 1000 possible sources. The mean amplitude of these known dictionary elements was one. (B–D) Adding an extra source that is not part of the dictionary with increasing standard deviation amplitude of 0.25, 0.5 and 1 causes the larger level of background activation in the presence parameters. However, the iCPA is robust to the presence of this “non-dictionary” element. (TIF) [file pone.0024270.s004.tif]

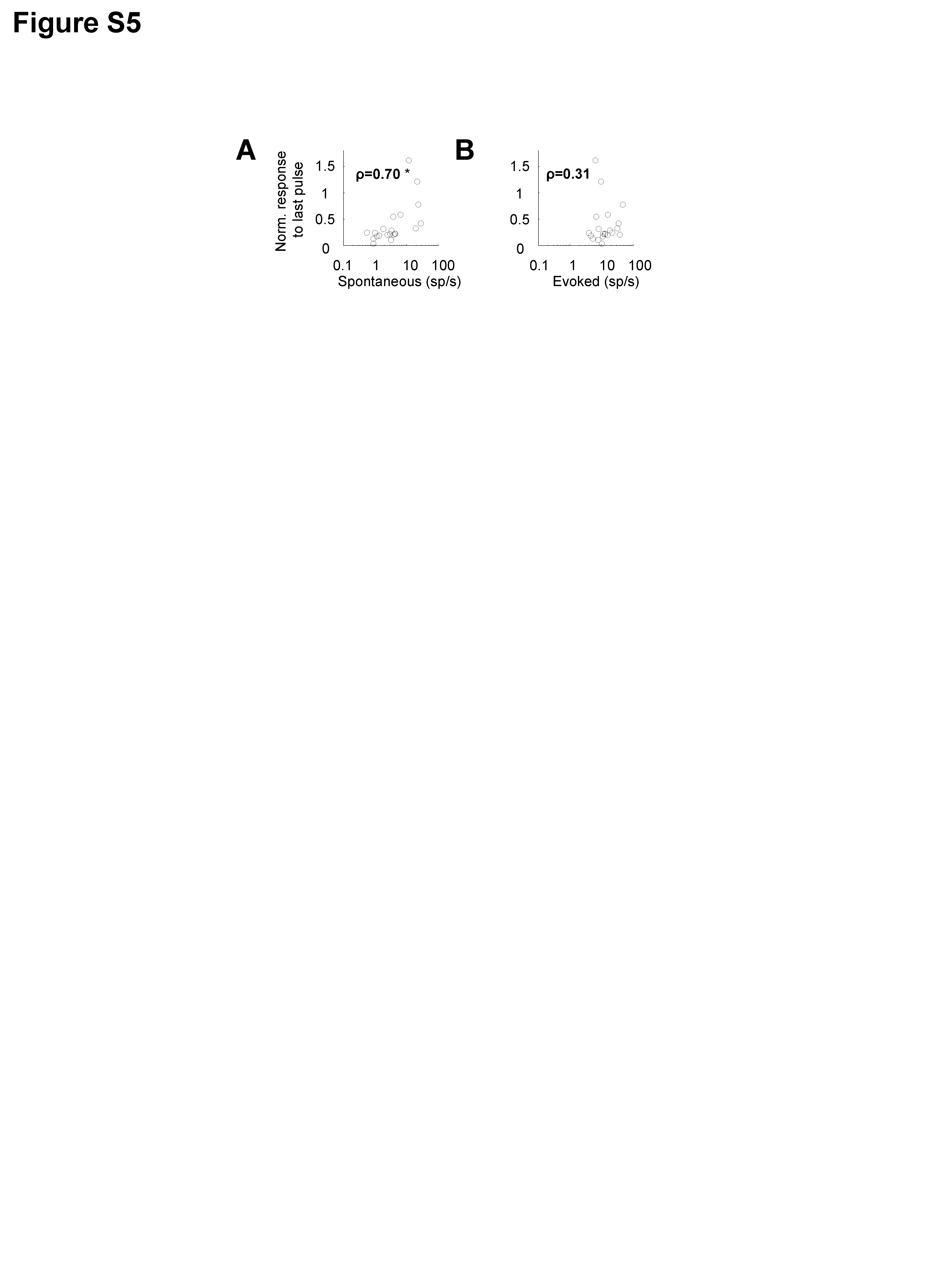

Supplement: Figure S5 — There was a correlation between spontaneous activity and normalized response of the last click for a 20 click per second train. (A) There is a significant correlation between the spontaneous firing rate and the normalized response of the last click of a 20 click/sec (B) There was no significant correlation between the normalized response of the last pulse and the response evoked by the first click (see Text S6: Statistics). (TIF) [file pone.0024270.s005.tif]

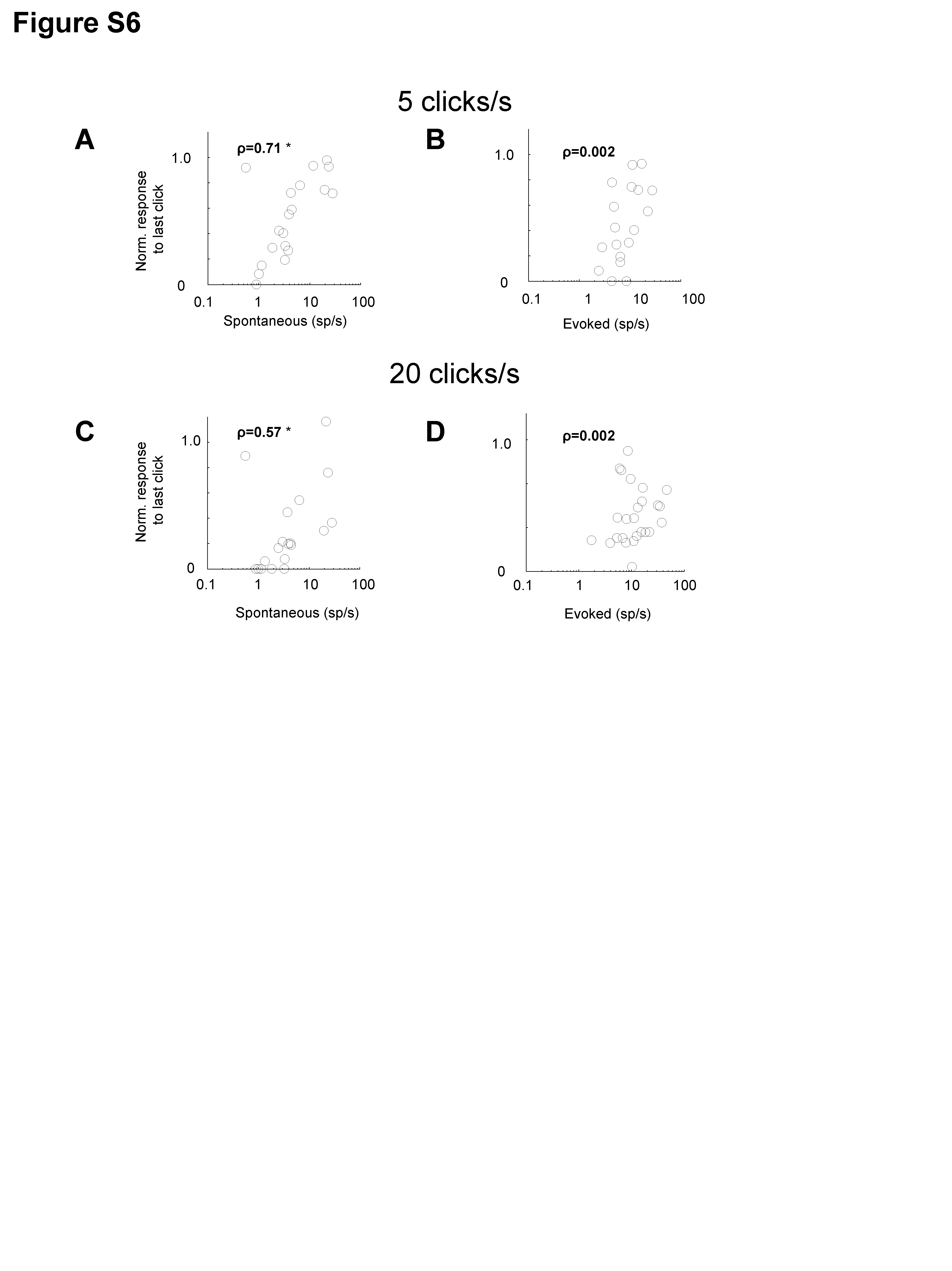

Supplement: Figure S6 — Correlations between normalized response of the last click and spontaneous activity was maintained after subtracting the spontaneous activity. There was a significant correlation between the spontaneous activity and the normalized response of the last click of (A) the 5 click/sec train and (C) the 20 clicks/sec train. The responses to the clicks were calculated by subtracting the spontaneous activity from the evoked response. There was no significant correlation between the spontaneous subtracted normalized response of the last click and the evoked activity for neither the 5 clicks/sec train (B) nor for the 20 clicks/sec train (D). (See Text S6: Statistics.) (TIF) [file pone.0024270.s006.tif]
