# Supplementary material for: A Corticothalamic Circuit Model for Sound Identification in Complex Scenes
Source: PLoS One. 2011 Sep 13;6(9):e24270. doi: 10.1371/journal.pone.0024270 (PMC3172241; doi:10.1371/journal.pone.0024270)
Supplement: Text S1 — Definition of auditory scene. (DOC) [file pone.0024270.s007.doc]

**Text S1: Definition of auditory scene**

We consider that the auditory scene is defined by its power spectrum at time *t*, represented by an *f*-dimensional vector$\vec{y}(t)$. This vector is assumed to arise from a linear combination of *n* dictionary elements. At time$t$, the scene$\vec{y}(t)$is composed of the dictionary elements$\vec{B}_{k}$ according to the proportions given by the time-varying coefficients $A^{k}(t),k=1,..,n$, such that we can write

S1. 1

The random variables $A^{k}(t)$ are assumed to be uncorrelated to each other,

$<A^{k}(t)A^{j}(t)>=\left\{\begin{matrix} 0,k\neq j \\ <(A^{k})^{2}>,k=j \end{matrix}\right\}$S1. 2

Here, the brackets <> indicate averages over time.

We can write$\vec{y}(t)$ as an *f*-dimensional column vector and combine the$A^{k}(t)$ into an *n*-dimensional column vector as

$\vec{y}(t)=\left[\begin{matrix} y^{1}(t) \\ y^{2}(t) \\ \vdots\\ y^{f}(t) \end{matrix}\right]$S1. 3

$\vec{A}(t)=\left[\begin{matrix} A^{1}(t) \\ A^{2}(t) \\ \vdots\\ A^{n}(t) \end{matrix}\right]$S1. 4

Each $\vec{B}_{k}$ can also be written as an *f*-dimensional column vector:

$\vec{B}_{k}=\left[\begin{matrix} B_{k}^{1} \\ B_{k}^{2} \\ \vdots\\ B_{k}^{f} \end{matrix}\right]$S1. 5

If we combine all the individual vectors $\vec{B}_{k}$ into an *f* by *n* matrix:

S1. 6

the signal $\vec{y}(t)$ can be expressed as the matrix multiplication

S1. 7

Although the dictionary **B** has *n* vectors, any particular auditory scene is assumed to be generated by linear combinations of only a small set of the dictionary elements, that is, most of the time averages $<(A^{k})^{2}>$are zero.
