# Supplementary material for: A Corticothalamic Circuit Model for Sound Identification in Complex Scenes
Source: PLoS One. 2011 Sep 13;6(9):e24270. doi: 10.1371/journal.pone.0024270 (PMC3172241; doi:10.1371/journal.pone.0024270)
Supplement: Text S2 — Corrected projections algorithm. (DOC) [file pone.0024270.s008.doc]

**Text S2: Corrected projections algorithm**

The corrected projections algorithm (CPA) is a method for identifying the dictionary elements that are present in an auditory scene$\vec{y}(t)$. The method is based on constructing an estimate $\hat{y}(t)$ that minimizes the error between $\vec{y}(t)$ and $\hat{y}(t)$ in the least mean square sense for *T* observations of the signal $\vec{y}(t)$, *t=1,..,T*. To obtain the estimate$\hat{y}(t)$, we try to fit parameters $\theta_{k},k=1,..,n$ according to the model

S2. 1

The weighting factors of the dictionary elements $\vec{B}_{k}$consist of the scalar products and the parameters $\theta_{k}$ that minimize the error. The projections $\vec{\phi}_{k}(t)$ of the vector $\vec{y}(t)$ into the dictionary element$\vec{B}_{k}$are then defined as a vector in the direction of the dictionary element$\vec{B}_{k}$, whose magnitude is proportional to the scalar product,

S2. 2

To simplify notation, we arrange the *T* observations $\vec{y}(t)$ and the *T* estimates $\hat{y}(t)$ into  *(T x f)*-dimensional column vectors:

and S2. 3

If we then arrange the projections $\vec{\phi}_{k}(t)$ into a *(T x f) by n* matrix

S2. 4

and write the factors$\theta_{k}$ as an *n*-dimensional column vector called $\vec{\Theta}$

$\vec{\Theta}=\left[\begin{matrix} \theta_{1} \\ \theta_{2} \\ \vdots\\ \theta_{n} \end{matrix}\right]$S2. 5

then the estimate $\hat{Y}$is given by the matrix multiplication:

$\hat{Y}=\mathbf{\Phi} \vec{\Theta}$S2. 6

We would like to find the vector $\vec{\Theta}$ that will minimize the mean square error between the observation *Y* and the estimate $\hat{Y}$. This is a linear least squares problem.

The vector $\vec{\Theta}$ can be calculated by (Astrom and Wittenmark 1995):

$\vec{\Theta}=(\mathbf{\Phi}^{tr}\mathbf{\Phi})^{-1}\mathbf{\Phi}^{tr} Y$ S2. 7

under the condition that the matrix$(\mathbf{\Phi}^{tr}\mathbf{\Phi})$has an inverse. For most practical purposes, the matrix$(\mathbf{\Phi}^{tr}\mathbf{\Phi})$ is invertible, as we will show next.

The elements of matrix $(\mathbf{\Phi}^{tr}\mathbf{\Phi})$ are as follows:

S2. 8

When the number of dimensions *f* of the signal $\vec{y}(t)$ is large, then the dot products

, if $i\neq j$. S2. 9

are small (see **Text S4** for more details). Therefore, the matrix $(\mathbf{\Phi}^{tr}\mathbf{\Phi})$ is almost diagonal because the off-diagonal elements depend on this very small value. The inverse of a diagonal matrix exists if the diagonal elements are not zero. The diagonal elements are given by:

S2. 10

Therefore, for the inverse to exist, it is also necessary that the vectors considered $\vec{B}_{i}$ are not orthogonal to all the *T* samples of the signal $\vec{y}(t)$, that is, for a given vector $\vec{B}_{i}$:

**S2. 11**

for at least one of the samples *t=1,..,T*.

Nevertheless, there are situations, e.g., $\vec{y}(t)=0$ (a standard initial condition), in which the matrix $(\mathbf{\Phi}^{tr}\mathbf{\Phi})$ does not have an inverse. In that case, we could use the solution

$\vec{\Theta}=\mathop{[\lim}\limits_{\delta\to0} (\delta\mathbf{I}+\mathbf{\Phi}^{tr}\mathbf{\Phi})^{-1}\mathbf{\Phi}^{tr}] Y$S2. 12

where the brackets on the right-hand side denote the Moore-Penrose inverse of $\mathbf{\Phi}$and ***I*** is the unit matrix. The Moore-Penrose inverse exists even if the matrix$(\mathbf{\Phi}^{tr}\mathbf{\Phi})$has no inverse. The equation **S2.12** yields the solution, e.g., $\vec{\Theta}=0$ , in the case of $\vec{y}(t)=0$.

Astrom, K. J. and B. Wittenmark (1995). "Adaptive Control, Second Edition." Prentice Hall.
