# Supplementary material for: A Corticothalamic Circuit Model for Sound Identification in Complex Scenes
Source: PLoS One. 2011 Sep 13;6(9):e24270. doi: 10.1371/journal.pone.0024270 (PMC3172241; doi:10.1371/journal.pone.0024270)
Supplement: Text S3 — Proof that CPA detects the elements present in a mixture. (DOC) [file pone.0024270.s009.doc]

**Text S3: Proof that CPA detects the elements present in an auditory scene**

$\hat{y}(t)$To prove that CPA actually detects the elements present, we will express the parameters $\theta_{k},k=1,..,n$ obtained by CPA in responses to a signal$\vec{y}(t)$ as functions of the parameters $A^{k}(t),k=1,..,n$ that were used to generate this signal (**Text S1: Definition of auditory scene**). To this end, we define the average error $<V>$ in the reconstruction as

S3. 1

where the brackets indicate an average over time.

The corrected projection algorithm, calculated using **S2.7**, finds parameters $\theta_{k}$ that minimize the average error $<V>$. If we write the parameters $\theta_{k}$in a diagonal matrix

$\mathbf{\Theta}=\left[\begin{matrix} \theta_{1} \\ 0 \\ \vdots\\ 0 \end{matrix}\begin{matrix} 0 \\ \theta_{2} \\ \vdots\\ 0 \end{matrix}\begin{matrix} ... \\ ... \\ \ddots\\ ... \end{matrix}\begin{matrix} 0 \\ 0 \\ \vdots\\ \theta_{n} \end{matrix}\right]$ S3. 2

the estimate $\hat{y}(t)$ can be calculated as a matrix multiplication, using matrix **B** defined in **S1.6** as

S3. 3

Inserting the estimator $\hat{y}(t)$ into **S3.1**, we obtain

S3. 4

The input $\vec{y}(t)$ in **S3.4** can be replaced by its components, as expressed in **S1.7**:

S3. 5

such that equation **S3.4** becomes

S3. 6

If we rewrite this expression to separate $\vec{A}(t)$ from the estimated parameters, we obtain

S3. 7

or

S3. 8

where

$\mathbf{M}=(\mathbf{I}-\mathbf{B}^{tr}\mathbf{B}\Theta)\mathbf{B}^{tr}\mathbf{B}(\mathbf{I}-\Theta\mathbf{B}^{tr}\mathbf{B})$ S3. 9

and **I** is the *n by n* unit matrix.

The matrix multiplications in **S3.8** can be written in terms of ***M*** as

$\begin{matrix} <V>=<A^{1}(t)[A^{1}(t) M_{1,1}+A^{2}(t) M_{1,2}+...A^{n}(t) M_{1,n}]+ \\ A^{2}(t) [A^{1}(t) M_{2,1}+A^{2}(t) M_{2,2}+...A^{n}(t) M_{2,n}]+ \\ ... \\ A^{n}(t) [A^{1}(t) M_{n,1}+A^{2}(t) M_{n,2}+...A^{n}(t) M_{n,n}]> \end{matrix}$ S3. 10

We can split up this expression between the diagonal elements of ***M*** and the outer-diagonal terms of ***M***:

$\begin{matrix} <V>=<\sum_{i=1}^{n} (A^{i}(t))^{2} M_{i,i}+\sum_{i=1}^{n} \sum_{j=1,j\neq i}^{n} A^{i}(t) A^{j}(t) M_{i,j}> \\ =\sum_{i=1}^{n} <(A^{i})^{2}> M_{i,i}+\sum_{i=1}^{n} \sum_{j=1,j\neq i}^{n} <A^{i}A^{j}> M_{i,j} \end{matrix}$S3. 11

Since the cross-correlation of the coefficients $A^{k}(t)$ is zero (see **S1.2**), the average error depends only on the diagonal elements $M_{i,i}$of the matrix ***M***.

$<V>=\sum_{i=1}^{n} <(A^{i})^{2}> M_{i,i}$S3. 12

To compute the diagonal elements of **M**, we use the following expression

$\mathbf{M}=(\mathbf{I}-\mathbf{C\Theta})\mathbf{C}(\mathbf{I}-\mathbf{\Theta C})=\mathbf{C}-2\mathbf{C\Theta}C+\mathbf{C\Theta}C\mathbf{\Theta C}$ S3. 13

in which

S3. 14

The matrix elements of **C** are scalar products

S3. 15

and hence ***C*** is symmetric. Based on **S3.13**, the diagonal elements $\mathbf{M}_{i,i}$can thus be expressed as

$M_{i,i}=c_{i,i}-2\sum_{k=1}^{n} c_{i,k}\theta_{k}c_{k,i}+\sum_{h=1}^{n} c_{i,h}\theta_{h}\sum_{k=1}^{n} c_{h,k}\theta_{k}c_{k,i}$ **S3. 16**

To find the parameters $\theta_{k},k=1,..,n$ that minimize the error$<V>$, we have to compute the derivative of $M_{i,i}$ with respect to the parameters$\theta_{l}$,

$\frac{\partial M_{i,i}}{\partial\theta_{l}}=-2c_{i,l}c_{l,i}+c_{i,l} \sum_{k=1}^{n} c_{l,k}\theta_{k}c_{k,i}+c_{l,i} \sum_{h=1}^{n} c_{i,h}\theta_{h}c_{h,l}$ S3. 17

Changing the index in the second sum from *h* to *k*, we obtain

$\frac{\partial M_{i,i}}{\partial\theta_{l}}=-2(c_{i,l})^{2}+2c_{i,l}\sum_{k=1}^{n} c_{l,k}\theta_{k}c_{k,i}$ **S3. 18**

By taking the derivative of *<V>* respect to$\theta_{l}$ in **S3.12** and making it equal to zero, we get

$\begin{matrix} \frac{d<V>}{d\theta_{l}}=\sum_{i=1}^{n} <(A^{i})^{2}> \frac{dM_{i,i}}{d\theta_{l}}=0 \\ l=1,..,n \end{matrix}$ S3. 19

We can write this expression in terms of the matrices ***C*** and $\mathbf{\Theta}$, by placing **S3.18** in **S3.19**

$\sum_{i=1}^{n} <(A^{i})^{2}>(c_{i,l})^{2}=\sum_{i=1}^{n} \sum_{k=1}^{n} c_{l,i}<(A^{i})^{2}>c_{i,k}\theta_{k}c_{k,l}$ , *l = 1,..n* S3. 20

The solutions$\theta_{k}$,*k=1,..,n* of this system of *n* equations define a set of parameters $\theta_{k}$ that minimizes the error in the estimation.

A specific assumption was that each particular auditory scene is composed of only a few elements. Therefore, there is a group of dictionary elements$\vec{B}_{k}$, which we define as the active set, meaning

$<(A^{k})^{2}>=O(1)\neq0$ for $k\in A$ S3. 21

and a non-active set

$<(A^{k})^{2}>=0$ for $k\notin A$ S3. 22

where $A\subset\{1,2,...n\}$ denotes the set of dictionary elements that contribute to the current auditory scene. As two of the sums in **S3.20** (indexed by *i*) run over the average power $<(A^{i})^{2}>$, we can restrict those to the active set, and hence write

$\sum_{i\in A} <(A^{i})^{2}> (c_{i,l})^{2}=\sum_{i\in A} c_{l,i}<(A^{i})^{2}>\sum_{k=1}^{n} c_{i,k}\theta_{k}c_{k,l},l=1..n$ **S3. 23**

The goal of CPA is to determine the *n* variables$\theta_{l}$, *l=1,...,n* that solve for the *n* linear equations described in **S3.23**. To do so, we will first find an exact solution for a situation where the dot products between the dictionary elements that are present in a scene are zero,

, if $i,l\in A,i\neq l$ **S3. 24**

that is, simultaneously present sources are orthogonal. Later on, in **Text S4**, we will show that, if the dimension *f* of the dictionary element vectors is large enough, the actual solutions are close to the exact solution of the orthogonal case, even if the sources present are not strictly orthogonal.

To derive the exact solution of **S3.23** under condition **S3.24**, we will, first, consider elements that belonged to the auditory scene and then we will consider the elements that were not part of the active set.

**Case 1:** $l\in A$, i.e., Element *l* took part in the auditory scene, $<(A^{l})^{2}>\neq0$.

For the sums over$i\in A$ in **S3.23,** we separate the terms for *i=l* and$i\neq l$,

$\begin{matrix} <(A^{l})^{2}>(c_{l,l})^{2}+\sum_{i\in A,i\neq l} <(A^{i})^{2}> (c_{i,l})^{2}=c_{l,l}<(A^{l})^{2}> \left(c_{l,l}\theta_{l}c_{l,l}+\sum_{k=1,k\neq l}^{n} \theta_{k}(c_{k,l})^{2}\right) \\ +\sum_{i\in A,i\neq l} c_{l,i}<(A^{i})^{2}> \left(c_{i,l}\theta_{l}c_{l,l}+\sum_{k=1,k\neq l}^{n} c_{i,k}\theta_{k}c_{k,l}\right) \end{matrix}$ **S3. 25**

Considering that$c_{l,l}=1$, and splitting up the remaining sums into active and inactive dimensions, expression **S3.25** becomes

$\begin{matrix} <(A^{l})^{2}>+\sum_{i\in A,i\neq l} <(A^{i})^{2}> (c_{l,i})^{2}=<(A^{l})^{2}> \left(\theta_{l}+\sum_{k\in A,k\neq l} \theta_{k}(c_{k,l})^{2}+\sum_{k\notin A} \theta_{k}(c_{k,l})^{2}\right) \\ +\sum_{i\in A,i\neq l} c_{l,i}<(A^{i})^{2}> \left(c_{i,l}\theta_{l}+\sum_{k\in A,k\neq l} c_{i,k}\theta_{k}c_{k,l}+\sum_{k\notin A} c_{i,k}\theta_{k}c_{k,l}\right) \end{matrix}$ **S3. 26**

Since we have assumed the orthogonality of the active dictionary elements, **S3.26** simplifies to

$<(A^{l})^{2}>=<(A^{l})^{2}> \left(\theta_{l}+\sum_{k\notin A} \theta_{k} (c_{k,l})^{2}\right)$ **S3. 27**

Equation **S3.27** is evidently solved for $\theta_{l}=1, l\in A$and$\theta_{k}=0, k\notin A$. Next, we will show that this also solves the equations for inactive dimensions (see Case 2).

**Case 2: $l\notin A$,** i.e., element *l* did not take part in the auditory scene, $<(A^{l})^{2}>=0$.

Again, by separating the active and inactive indices, we write **S3.23** as

$\sum_{i\in A} <(A^{i})^{2}> (c_{i,l})^{2}=\sum_{i\in A} c_{l,i}<(A^{i})^{2}> \left(c_{i,l}\theta_{l}+\sum_{k\in A} c_{i,k}\theta_{k}c_{k,l}+\sum_{k\notin A,k\neq l} c_{i,k}\theta_{k}c_{k,l}\right)$ **S3. 28**

Applying the orthogonality condition of the active elements, **S3.28** becomes

$\sum_{i\in A} <(A^{i})^{2}> (c_{i,l})^{2}=\sum_{i\in A} c_{l,i}<(A^{i})^{2}> \left(c_{i,l}\theta_{l}+c_{i,i} \theta_{i} c_{i,l}+\sum_{k\notin A,k\neq l} c_{i,k}\theta_{k}c_{k,l}\right)$ **S3. 29**

Inserting the Ansatz $\theta_{l}=1, l\in A$and$\theta_{k}=0, k\notin A$ yields the identity

$\sum_{i\in A} <(A^{i})^{2}> (c_{i,l})^{2}=\sum_{i\in A} c_{l,i}<(A^{i})^{2}> c_{i,l}$ **S3. 30**

Therefore, we have shown that **S3.23**, and hence the minimization problem **S3.4**, is solved if the presence parameter $\theta_{l}$for an element $\vec{B}_{l}$ that is part of the active set is equal to one and if the presence parameter $\theta_{l}$for an element $\vec{B}_{l}$ that is not part of the active set is equal to zero.
