# Supplementary material for: A Corticothalamic Circuit Model for Sound Identification in Complex Scenes
Source: PLoS One. 2011 Sep 13;6(9):e24270. doi: 10.1371/journal.pone.0024270 (PMC3172241; doi:10.1371/journal.pone.0024270)
Supplement: Text S4 — Effects of auditory scene complexity and dictionary size on CPA performance. (DOC) [file pone.0024270.s010.doc]

**Text S4: Effects of auditory scene complexity and dictionary size on CPA performance**

CPA outputs parameters $\theta_{k}=1$ if an element $\vec{B}_{k}$ was part of an auditory scene and outputs $\theta_{k}=0$ if it was not part of that auditory scene. The solutions are exact under the assumption that the scalar products between the dictionary elements that were present in a particular auditory scene were zero, that is:

, S4. 1

if

$<(A^{k})^{2}>,<(A^{l})^{2}>\neq0$ **. S4. 2**

The maximal number *n* of dictionary elements, such as that they are mutually orthogonal, is *f*, the number of dimensions of the input vectors. It is possible to have a much larger dictionary of *n* (*n>>f*) elements, if the values of the estimated parameters $\theta_{k}$ were robust against the dot products having small values $c_{k,l}=O(\varepsilon), \varepsilon<<1$. In this section, we will show that, if the number of dimensions *f* is large enough, the dictionary elements will be approximately orthogonal and the algorithm is still valid.

We start by perturbing the solution of section **Text S3** by adding small deviations $\rho, \sigma$that converge to zero for$\varepsilon\to0$:

$\theta_{l}=1+\rho_{l}$, if $l\in A$, S4. 3

and

$\theta_{l}=0+\sigma_{l}$, if $l\notin A$ S4. 4

These small parameters measure the deviation of the estimated parameters from the unperturbed solution, when $c_{k,l}\neq0$. The purpose of the following derivation is to express the perturbation factors $\rho$ and $\sigma$in terms of $c_{k,l}\neq0$.

Inserting **S4.3** and **S4.4** into **S3.23** and again separating into active and inactive indices, we get two equations. For inactive indices$l\notin A$, we find

$\begin{matrix} \sum_{i\in A} <(A^{i})^{2}> (c_{i,l})^{2}=\sum_{i\in A} c_{l,i}<(A^{i})^{2}> \left(\sum_{k\in A} c_{i,k}(1+\rho_{k})c_{k,l}+\sum_{k\notin A,} c_{i,k}\sigma_{k}c_{k,l}\right) \\ =\sum_{i\in A} (c_{l,i})^{2}<(A^{i})^{2}> (1+\rho_{i}) +\sum_{i\in A} \sum_{k\in A,k\neq i} c_{l,i}<(A^{i})^{2}> c_{i,k}(1+\rho_{k}) c_{k,l} \\ +\sum_{i\in A} \sum_{k\notin A} c_{l,i}<(A^{i})^{2}> c_{i,k}\sigma_{k} c_{k,l} \end{matrix}$ S4. 5

Similarly, we can derive an equation for $l\in A$:

$\begin{matrix} <(A^{l})^{2}>+\sum_{i\in A,i\neq l} <(A^{i})^{2}> (c_{l,i})^{2}=<(A^{l})^{2}> \left(1+\rho_{l}+\sum_{k\in A,k\neq l} (1+\rho_{k})(c_{k,l})^{2}+\sum_{k\notin A} \sigma_{k}(c_{k,l})^{2}\right) \\ +\sum_{i\in A,i\neq l} c_{l,i}<(A^{i})^{2}> \left(c_{i,l}(1+\rho_{l})+\sum_{k\in A,k\neq l} c_{i,k}(1+\rho_{k})c_{k,l}+\sum_{k\notin A} c_{i,k}\sigma_{k}c_{k,l}\right) \end{matrix}$ S4.6

A part of the left-hand side cancels out such that

$\begin{matrix} \sum_{i\in A,i\neq l} <(A^{i})^{2}> (c_{l,i})^{2}=<(A^{l})^{2}> \left(\rho_{l}+\sum_{k\in A,k\neq l} (1+\rho_{k})(c_{k,l})^{2}+\sum_{k\notin A} \sigma_{k}(c_{k,l})^{2}\right) \\ +\sum_{i\in A,i\neq l} c_{l,i}<(A^{i})^{2}> \left(c_{i,l}(1+\rho_{l})+\sum_{k\in A,k\neq l} c_{i,k}(1+\rho_{k})c_{k,l}+\sum_{k\notin A} c_{i,k}\sigma_{k}c_{k,l}\right) \end{matrix}$ S4. 7

Similarly as for the unperturbed case in **Text S3**, we will now construct a self-consistent solution of **S4.5** and **S4.7**. To this end, we will assume that the deviations are small,$\rho<<1, \sigma<<1$. Furthermore, we assume that the scalar products *c* are small enough such that

$\sum_{i\in A,i\neq l} c_{l,i}=O(\sqrt{p-1} \varepsilon)<<1$, S4. 8

where *p* denotes the number of active elements, or equivalently, we assume that the number of active elements is bounded$p<<1/\varepsilon^{2}$. The notation *O( )* indicates the order of magnitude of a variable.

Using the assumption that $\rho<<1$, we can approximate

$(1+\rho_{k})c_{k,l}\approx c_{k,l}$ S4. 9

We can use this approximation to simplify **S4.7** to obtain

$\begin{matrix} \sum_{i\in A,i\neq l} <(A^{i})^{2}>\left(c_{l,i}\right)^{2}=<(A^{l})^{2}> \left(\rho_{l}+\sum_{k\in A,k\neq l} (c_{k,l})^{2}+\sum_{k\notin A} \sigma_{k}(c_{k,l})^{2}\right) \\ +\sum_{i\in A,i\neq l} c_{l,i}<(A^{i})^{2}> \left(c_{i,l}+\sum_{k\in A,k\neq l} c_{i,k}c_{k,l}+\sum_{k\notin A} c_{i,k}\sigma_{k}c_{k,l}\right) \end{matrix}$ S4. 10

The left side of **S4.10** is canceled out as

$\begin{matrix} 0=<(A^{l})^{2}> \left(\rho_{l}+\sum_{k\in A,k\neq l} (c_{k,l})^{2}+\sum_{k\notin A} \sigma_{k}(c_{k,l})^{2}\right) \\ +\sum_{i\in A,i\neq l} c_{l,i}<(A^{i})^{2}> \left(\sum_{k\in A,k\neq l} c_{i,k}c_{k,l}+\sum_{k\notin A} c_{i,k}\sigma_{k}c_{k,l}\right) \end{matrix}$ S4. 11

The elements of the sums in the first term are $(c_{k,l})^{2}$, the square of the dot products, whereas the elements of the sums in the second term are $c_{i,k}c_{k,l}$, making the elements of the second term much smaller than the elements of the first term. Therefore, we can approximate $\rho_{l}$ as

$\rho_{l}=-\sum_{k\in A,k\neq l} (c_{k,l})^{2}-\sum_{k\notin A} \sigma_{k}(c_{k,l})^{2}$ **S4. 12**

The order of magnitude is estimated according to the central limit theorem, yielding:

$\rho_{l}=O((p-1) \varepsilon^{2})+O(\sqrt{n} \varepsilon^{2} \sigma)$ **S4. 13**

We can apply the same assumptions to equation **S4.5** for the inactive index set. Applying **S4.9**, we get

$\begin{matrix} \sum_{i\in A} <(A^{i})^{2}> (c_{i,l})^{2}=\sum_{i\in A} (c_{l,i})^{2}<(A^{i})^{2}>+\sum_{i\in A} \sum_{k\in A,k\neq i} c_{l,i}<(A^{i})^{2}> c_{i,k}c_{k,l} \\ +\sum_{i\in A} \sum_{k\notin A} c_{l,i}<(A^{i})^{2}> c_{i,k}\sigma_{k} c_{k,l} \end{matrix}$ **S4. 14**

By canceling out the left-side term, we obtain

$0=\sum_{i\in A} \sum_{k\in A,k\neq i} c_{l,i}<(A^{i})^{2}> c_{i,k}c_{k,l}+\sum_{i\in A} \sum_{k\notin A} c_{l,i}<(A^{i})^{2}> c_{i,k}\sigma_{k} c_{k,l}$ **S4. 15**

We can separate from the summations the terms that depend on *k=l* as

$\begin{matrix} 0=\sum_{i\in A} \sum_{k\in A,k\neq i} c_{l,i}<(A^{i})^{2}> c_{i,k}c_{k,l}+\sum_{i\in A} \sum_{k\notin A,k\neq l} c_{l,i}<(A^{i})^{2}> c_{i,k}\sigma_{k} c_{k,l}+ \\ +\sigma_{l}\sum_{i\in A} <(A^{i})^{2}>\left(c_{i,l}\right)^{2} \end{matrix}$ **S4. 16**

Again, the order of magnitude is estimated according to the central limit theorem, yielding:

$0=O(\sqrt{p(p-1) } \varepsilon^{3})+O(\sqrt{pn} \varepsilon^{3}\sigma)+O(p \varepsilon^{2}\sigma)$ **S4. 17**

A condition that the number *n* of dictionary elements is large enough, and that the auditory scenes are composed of a few elements *p*, would imply:

$\varepsilon\sqrt{n}>>\sqrt{p}$ **S4. 18**

Therefore

$O(\sqrt{pn} \varepsilon^{3}\sigma)>>O(p \varepsilon^{2}\sigma)$, **S4. 19**

and **S4.17** becomes

$0=O(\sqrt{p(p-1) } \varepsilon^{3})+O(\sqrt{pn} \varepsilon^{3}\sigma)$ **S4. 20**

We can conclude then that

$O(\sigma)=\sqrt{\frac{p-1}{n}}$ **S4. 21**

If we replace **S4.21** in **S4.13**, we obtain

$\rho_{l}=O((p-1) \varepsilon^{2})+O(\sqrt{n} \varepsilon^{2}\sqrt{\frac{p-1}{n}})$ **S4. 22**

The dependency on n *on* the second term cancels out, and we obtain

$O(\rho)=(p-1) \varepsilon^{2}$ **S4. 23**

We will now derive an expression for the dot product $\varepsilon$ as a function of the length *f* of the input signal$\vec{y}(t)$. In what follows, we assume that the entries of the dictionary elements $B_{k}^{i}$are statistically independent random variables with zero mean. The scalar product $c_{k,l},k\neq l$can be treated by the central limit theorem, meaning that the expected value is:

**S4. 24**

and the variance is given by:

$\mathrm{var}(c_{k,l})=f\mathrm{var}(B_{k}^{i} B_{l}^{i})=f\mathrm{var}(B_{k}^{i})\mathrm{var}(B_{l}^{i})=f\mathrm{var}(B_{k}^{i})^{2}$. **S4. 25**

This expression depends on the variance of the dictionary elements. Since the dictionary elements are normalized$\vec{B}_{k}$, that is,

, **S4. 26**

we can calculate the variance of the individual elements by:

. **S4. 27**

Therefore,

$\mathrm{var}(B_{k}^{i})=\frac{1}{f}$ **S4. 28**

By placing this expression into **S4.25**, we obtain

$\mathrm{var}(c_{k,l})=\frac{1}{f}$ **S4. 29**

We therefore approximate the distribution of the dot products *c* as the Gaussian distribution of zero mean and standard deviation$\varepsilon=f^{-1/2}$. Therefore, we can estimate the order of the deviations $\rho$ as

$O(\rho)=\frac{p-1}{\sqrt{f}}$ **S4. 30**

Equations **S4.21** and **S4.30** state that CPA is robust to deviations from orthogonality of the dictionary elements (see **Fig. 5A** for a simulation example for a signal of 400 dimensions and 68000 dictionary elements). Equation **S4.30** states that the deviations from the exact solution for the elements that took part in the auditory scene are small, if the number of dimensions *f* is large enough (see **Fig. 5D** for simulation results). The deviations also increase as the number of elements present in a scene *p* increases (see **Fig. 5C**).

Note that the derivation of this formula assumed that the distribution of$\sigma$has zero mean and therefore the order of magnitude of the sums in **S4.13** and **S4.17** scales like$\sqrt{n}$. If we assume a slight bias of$\sigma$, then the order of magnitude of$\rho$will increase with *n* (see **Fig. 5B**).
