# Supplementary material for: A Corticothalamic Circuit Model for Sound Identification in Complex Scenes
Source: PLoS One. 2011 Sep 13;6(9):e24270. doi: 10.1371/journal.pone.0024270 (PMC3172241; doi:10.1371/journal.pone.0024270)
Supplement: Text S5 — Recurrent implementation of CPA. (DOC) [file pone.0024270.s011.doc]

**Text S5: Derivation of iterative CPA**

The solution to the corrected projection method is calculated by

$\vec{\Theta}=(\mathbf{\Phi}^{tr}\mathbf{\Phi})^{-1}\mathbf{\Phi}^{tr}Y$S5. 1

where $\mathbf{\Phi}$is the matrix (*T x f) by n* matrix

S5. 2

Here, for the sake of a simple notation, we assume that the matrix $\mathbf{\Phi}^{T}\mathbf{\Phi}$has an inverse, which is the case for the large enough *f* number of dimensions (see **Text S2**). Nevertheless, the following derivation can be straightforwardly generalized by replacing the inverse with the infinitesimal definition of the Moore-Penrose pseudo inverse introduced in **Text S2**.

The derivation of a iterative algorithm is similar to the algebraic manipulations necessary to derive the Kalman filter equations. For an example, see chapter 2 of (Astrom and Wittenmark 1995).

The matrix$\mathbf{\Phi}$ is updated every time that a measurement $\vec{y}(t)$ is made, and a set of projections into the dictionary B () is calculated. We can write the projections at time *t* into an *f by n* matrix that we call$\phi(t)$:

S5. 3

We can rewrite the matrix $\mathbf{\Phi}$ generated by *T* measurements as the *(T x f) by n* matrix:

$\mathbf{\Phi}=\left[\begin{matrix} \phi(1) \\ \phi(2) \\ \vdots\\ \phi(T) \end{matrix}\right]$S5. 4

The *n by n* matrix ***P*** after *T* measurements is defined as:

$\mathbf{P}(T)=(\mathbf{\Phi}^{tr}\mathbf{\Phi})^{-1}=\left(\sum_{t=1}^{T} \phi^{tr}(t)\phi(t)\right)^{-1}$S5. 5

which means that

$\mathbf{P}^{-1}(T)=\mathbf{P}^{-1}(T-1)+\phi^{tr}(T)\phi(T)$ S5. 6

The estimate of the parameters after *T* measurements is

S5. 7

where$\vec{y}(t)$ is a *f-*dimensional column vector and *Y* is an *(T x f)*-dimensional vector (see **S2.3**).

By expanding the summation in **S5.7**, we obtain

S5. 8

We will now write the estimate after *T* measurements $\vec{\Theta}(T)$ as a function of the previous estimate (after *T-1* measurements) $\vec{\Theta}(T-1)$.

The estimated parameters at time *T-1* was

S5. 9

By multiplying both sides by the inverse of the square matrix ***P****(T-1)*, we obtain

S5. 10

By replacing **S5.6** into **S5.10**, we obtain

S5. 11

By replacing **S5.11** into **S5.8**, we obtain

S5. 12

This equation can be simplified into:

S5. 13

Simplifying this expression, we obtain:

S5. 14

which can be written as:

S5. 15

where ***K****(T)* is the *n by f* matrix:

$\mathbf{K}(T)=\mathbf{P}(T)\phi^{tr}(T)$S5. 16

We also need to be able to estimate ***P****(T)* based on the elements ***P****(T-1)*.

By taking the inverse of equation **S5.6**, we obtain

$\mathbf{P}(T)=(\mathbf{P}^{-1}(T-1)+\phi^{tr}(T)\phi(T))^{-1}=(\mathbf{P}^{-1}(T-1)+\phi^{tr}(T)\mathbf{I}\phi(T))^{-1}$ S5. 17

where ***I*** is an identity matrix of *f* by *f* dimensions.

Using the property of matrix inversion

$(\mathbf{A}+\mathbf{BCD})^{-1}=\mathbf{A}^{-1}-\mathbf{A}^{-1}\mathbf{B}(\mathbf{C}^{-1}+\mathbf{DA}^{-1}\mathbf{B})^{-1}\mathbf{DA}^{-1}$S5. 18

and defining

$\begin{matrix} \mathbf{A}=\mathbf{P}^{-1}(T-1) \\ \mathbf{B}=\phi^{tr}(T) \\ \mathbf{C}=\mathbf{I} \\ \mathbf{D}=\phi(T) \end{matrix}$S5. 19

we can therefore write **S5.17** as:

$\mathbf{P}(T)=\mathbf{P}(T-1)-\mathbf{P}(T-1)\phi^{tr}(T)(\mathbf{I}+\phi(T)\mathbf{P}(T-1)\phi^{tr}(T))^{-1}\phi(T)\mathbf{P}(T-1)$S5. 20

We can summarize the algorithm as:

S5. 21

$$\mathbf{K}(T)=\mathbf{P}(T)\phi^{tr}(T)$$

$$\mathbf{P}(T)=\mathbf{P}(T-1)-\mathbf{P}(T-1)\phi^{tr}(T)(\mathbf{I}+\phi(T)\mathbf{P}(T-1)\phi^{tr}(T))^{-1}\phi(T)\mathbf{P}(T-1)$$

where

.

The recurrent implementation requires the inversion of the *f by f* matrix $(\mathbf{I}+\phi(T)\mathbf{P}(T-1)\phi^{T}(T))$ . The non-recurrent implementation requires the inversion of an *n by n* matrix. In the case where the number *n* of vectors of the dictionary is much larger than the dimensions of the signal *f*, it simplifies calculations, in terms of computational complexity as well as in numerical stability.

We can use the approximation

$$(\mathbf{I}+x)^{-1}\approx(\mathbf{I}-x)$$

to simplify the expression for $\mathbf{P}(T)$

$\mathbf{P}(T)=\mathbf{P}(T-1)-\mathbf{P}(T-1)\phi^{tr}(T)(\mathbf{I}-\phi(T)\mathbf{P}(T-1)\phi^{tr}(T))\phi(T)\mathbf{P}(T-1)$ S5. 22

In this case, iterative CPA does not need a matrix inversion and the algorithm can be implemented using multiplication and additions, which are operations that can be performed by neural elements.

Astrom, K. J. and B. Wittenmark (1995). "Adaptive Control, Second Edition." Prentice Hall.
