# Supplementary material for: A Corticothalamic Circuit Model for Sound Identification in Complex Scenes
Source: PLoS One. 2011 Sep 13;6(9):e24270. doi: 10.1371/journal.pone.0024270 (PMC3172241; doi:10.1371/journal.pone.0024270)
Supplement: Text S6 — Statistics. (DOC) [file pone.0024270.s012.doc]

**Text S6: Statistics**

**Figure S5**

Similar correlations to the ones in **Fig. 7J-K** were obtained for the normalized response of the last pulse of a 20 click/sec train (1.8 sec., 36 pulses). Out of the same 32 cells, 21 cells could be fitted with less than 10% error with the decaying exponential function. The correlation between the spontaneous activity and the normalized response of the last pulse of the 20 click/s train was r=0.70, p=0.00043, with the 95% confidence interval of the correlation coefficient between 0.38 and 0.87. There was also no significant correlation between the evoked response to the first click of the train and the normalized response of the last pulse of the 20 click/s train was (r=0.31, p=0.17, with the 95% confidence interval of the correlation coefficient between -0.14 and 0.66).

**Figure S6**

The evoked responses to the clicks were calculated after the spontaneous activity was subtracted. We repeated the tests from **Fig. 7J-K** and **Fig. S5.**  In the case of the responses to the 5 clicks/s train, out of the 32 cells, 20 cells could be fitted with less than 10% error with the decaying exponential function. There was a significant correlation (r=0.71, p=0.0005) between the spontaneous activity and the normalized response of the last pulse, with the 95% confidence interval between 0.38 and 0.87. There was no significant correlation (r=-0.0015, p=0.99) between the spontaneous activity and the normalized response of the last pulse, with the 95% confidence interval between -0.44 and 0.44.

In the case of the responses to the 20 clicks/s train, out of the 32 cells, 19 cells could be fitted with less than 10% error with the decaying exponential function. There was a significant correlation (r=0.57, p=0.011) between the spontaneous activity and the normalized response of the last pulse. There was no significant correlation (r=-0.0017, p=0.99) between the spontaneous activity and the normalized response of the last pulse.
